# Assessment of physiological and electrochemical effects of a repurposed zinc dithiocarbamate complex on *Acinetobacter baumannii* biofilms

**DOI:** 10.1038/s41598-022-16047-z

**Published:** 2022-07-09

**Authors:** Qing Yang, Kayode Olaifa, Fartisincha P. Andrew, Peter A. Ajibade, Obinna M. Ajunwa, Enrico Marsili

**Affiliations:** 1 Biofilm Laboratory, Department of Chemical and Materials Engineering, School of Engineering and Digital Sciences, Nazarbayev University, 53 Kabanbay Batyr Avenue, Nur-Sultan, 01000 Kazakhstan; 2 Department of Science Laboratory Technology, Modibbo Adama University, Yola, Nigeria; 3 School of Chemistry and Physics, University of KwaZulu-Natal, Scottsville, Pietermaritzburg, South Africa; 4 Department of Microbiology, Modibbo Adama University, Yola, Nigeria

**Keywords:** Microbiology, Antimicrobials

## Abstract

*Acinetobacter baumannii* is an infectious agent of global proportion and concern, partly due to its proficiency in development of antibiotic resistance phenotypes and biofilm formation. Dithiocarbamates (DTC) have been identified as possible alternatives to the current antimicrobials. We report here the evaluation of several DTC-metal complexes against *A. baumannii* planktonic cells and biofilms. Among the DTC-metal complexes and DTCs tested, ZnL1 (*N*-methyl-1-phenyldithiocarbamato-S,S′ Zn(II)), originally designed as an antitumor agent, is effective against biofilm forming *A. baumannii.* A MIC value of 12.5 µM, comparable to that of Gentamicin (5 µM) was measured for planktonic cells in tryptic soy broth. Spectroscopy, microscopy and biochemical analyses reveal cell membrane degradation and leakage after treatment with ZnL1. Bioelectrochemical analyses show that ZnL1 reduces biofilm formation and decreases extracellular respiration of pre-formed biofilms, as corroborated by microscopic analyses. Due to the affinity of Zn to cells and the metal chelating nature of L1 ligand, we hypothesize ZnL1 could alter metalloprotein functions in the membranes of *A. baumannii* cells, leading to altered redox balance. Results indicate that the DTC-Zn metal complex is an effective antimicrobial agent against early *A. baumannii biofilms* under laboratory conditions.

## Introduction

Antimicrobial resistance (AMR) has become a global health challenge with the continuous emergence of resistant phenotypes^1^. Several resistance mechanisms have been identified in microorganisms, which could be inherent or acquired from other strains, especially among bacteria^2^. The formation of biofilm, microstructured microbial communities encased in self-produced polymeric matrix, facilitate genetic transfer and the acquisition of antimicrobial resistance traits^3^.

AMR traits in prokaryotes can be classified according to their mechanism. While some traits are observed in both planktonic and biofilm forms, others are characteristic of the biofilm phenotype. AMR in planktonic cells could be the result of activities of efflux pumps, cellular modification of drug target site or secretion of antibiotic-hydrolyzing enzymes, among other mechanisms. In addition, AMR in biofilms arise from extracellular polymeric substance (EPS), which enclose microorganisms and block, or inactivate antimicrobial agents before they can reach the cells^1,4^. A combination of both planktonic and biofilm AMR mechanisms have been observed in both laboratory and in vivo studies. Bacteria with highest tendencies to evolve into drug resistant strains have been termed as ESKAPE group, which include *Enterococcus faecium, Staphylococcus aureus, Klebsiella pneumoniae*, *Acinetobacter baumannii*, *Pseudomonas aeruginosa*, and *Enterobacter* spp.^3^. Notably the clinical relevance of these microorganisms can be tied to their AMR versatility, which leads to the development of multi-drug resistant phenotypes^5^.

In healthcare settings, infections such as meningitis, septicemias, pneumonia, and endocarditis^3,5^, among others, have been linked to *A. baumannii* strains. The use of conventional antibiotics as a means of controlling *A. baumannii* infections has been the main practice; however, this has been challenged by the reported development of resistance of *A. baumannii* strains to cephalosporins, aminoglycosides, tetracyclines, quinolones, rifampicin, and other antibiotics^1^. This is attributed to the fact that *A. baumannii* possesses an effective genetic resistance system encoded in multiple resistance gene islands^3^. Drug resistant *A. baumannii* strains increase nosocomial infections, in-hospital stays and patient mortality largely due to poor antibiotic management^2^. According to the US center for disease control (CDC), *A. baumannii* had in 2019 been designated as an urgent threat to public health^6^. The lack of established schemes to manage *A. baumannii* bacteremia has been an important challenge as well^2^. It is therefore pertinent to determine new therapeutic options to manage *A. baumannii* infections.

Dithiocarbamates (DTCs) are small organic molecules with strong metal chelating^7^. DTCs are sulphur containing synthetic compounds, in which the sulphur group is strongly nucleophilic providing electrons for stable covalent bonding, with the stability of the bonding making it important in medicinal chemistry^8^. Synthetic DTC have been commercialized for their antifungal, anticancer, antioxidants and antibacterial properties and a good example of a commercially available DTC-metal complex is the antifungal agent Zineb^®^. This is a zinc complex of ethylenebis (dithiocarbamate) ligand, with the formula C_4_H_6_N_2_S_4_Zn^9^. It is important to evaluate novel DTC-metal complexes as potential antimicrobial agents, in response to the global surge in antimicrobial resistance. Zinc is the second most abundant trace element after iron and has a key role in human and microbial cells making it a good fit for therapeutic use^10^. Dependent on concentration, zinc has also been identified as a good antimicrobial agent, as exposure of bacteria to concentrations of Zn up to 400 µM causes intoxication leading to alterations in the homeostasis of the transition metal ion and damages oxidative stress response mechanisms of the bacteria^11^. Recently designed and synthesized DTCs and their Zinc-DTC complexes were utilized in laboratory anti-cancer studies showing good antiproliferative activity against TK10, UACC62 and MC7 human cancer cell lines with a IC_50_ ranging from 14.28 to 22.74 µM^12–15^. In this work, we tested the antibacterial efficacies of several DTCs, particularly DTC-Zn complex, repurposing as an anti-bacterial and antibiofilm agent against *A. baumannii*, using biochemical, bioelectrochemical and microscopic techniques. The results support the application of DTC-Zn as a potent antibacterial agent.

## Materials and methods

### Materials

*Acinetobacter baumannii* ATCC 19606, a biofilm producing strain, was used in this study. The strain was sub-cultured and maintained on tryptic soy broth (TSB) and nutrient agar (NA), while Mueller Hinton agar (MHA) was used for agar well dilution drug screening. Other materials used, including dimethyl sulfoxide (DMSO), gentamicin (Gen), crystal violet, glacial acetic acid, absolute ethanol, KCl, dipotassium hydrogen sulphate, adenosine diphosphate, magnesium chloride, glutaraldehyde, glycerol, phenol red, TES (*N*-[tris(hydroxymethyl)methyl]-2-aminoethanesulfonic acid), HEPES, Mg(CH_3_CO_2_)2·4H_2_O, sucrose, C_14_H_24_N_2_O_10_ (EGTA), C_6_H_13_NO_2_ (EACA), and 2-hydroxy-1,4-naphthoquinone (2-HNQ), were obtained from Sigma Aldrich and Thermo Fischer Scientific. All reagents were of analytical grade and were prepared according to manufacturer’s directions.

Bioelectrochemistry experiments were conducted with Screen-Printed Carbon Electrodes (SPE Ref. C110) from Metrohm DropSens, Spain, with graphite working electrode (WE) of 4 mm diameter and surface area of 0.126 cm^2^, graphite auxiliary electrode and silver (Ag) pseudoreference electrode. Electrochemical cells (EC) of 10 mL volume with a working volume of 8 mL were used. The following earlier synthesized DTCs: *N*-methyl-1-phenyldithiocarbamate, phenylpiperazyldithiocarbamate, morpholinyldithiocarbamate, and 2-(((4-methoxyphenyl)amino)methyl)phenolyldithiocarbamate designated L1, L2, L3, and L4 respectively were used. The zinc complexes of the DTCs: *N*-methyl-1-phenyldithiocarbamato-S,S′ zinc(II), Phenylpiperazyldithiocarbamato-S,S′ zinc(II), Morpholinyldithiocarbamato-S,S′ zinc(II), and 2-(((4-methoxyphenyl)amino)methyl)phenolyldithiocarbamato-S,S′ zinc(II) designated ZnL1, ZnL2, ZnL3, and ZnL4, respectively, were also used. Stock dilutions (50 mM) of all DTCs and DTC-Zn complexes were made in DMSO and kept at room temperature until used.

### Preliminary susceptibility screening

Initial screening to determine the antibacterial activity of the test compounds was carried out using standard agar well diffusion method in MHA seeded with optical density 0.1 OD_600_ *A. baumannii* cells (equivalent to 10^6^ colony forming units per milliliters—cfu mL^−1^). Agar wells were bored aseptically on the agar using a sterile cork borer and 100 µL of DTC/DTC-Zn concentrations (50–400 µM) were placed in different marked wells. Plates were incubated for 24 h at 37 °C and zones of inhibition (ZOI) were measured in millimeters. All susceptibility tests and further viability tests were carried out according to standard protocols of the Clinical and Laboratory Standards Institute (CLSI).

### Bacterial viability tests and MIC determination

Freshly prepared overnight cultures of *A. baumannii* cells were used. Cells were adjusted to optical density 0.1 OD_600_ and prepared in nutrient broth with the introduction of varied concentrations (5–50 µM) of most inhibitory DTC/DTC-Zn complex as determined in the ZOI tests (ZnL1). Reduced concentrations compared to agar well assay were used based on the inhibitory activity in agar well diffusion. Control experiments were conducted with Gen (1–12.5 µM) and untreated cells. Bacterial viability was determined using microtiter plate assays in 48 well plates incubated for 24 h at 37 °C under static conditions. Growth was monitored at 600 nm using a Gen5 TM Microplate Reader and Imager Software (BioTek Instruments). Triplicate experiments were conducted to obtain independent biological replicates. After incubation, results were reported as mean ± standard deviation (SD). Following the standard broth microdilution assay, the minimal inhibitory concentrations (MICs) were determined as the least concentration at which no visible growth was observed^16^.

### Physiological effect of different concentrations of test DTC-Zn on planktonic cells of ***A. baumannii:*** cell leakage analyses

To determine the influence of test DTC-Zn complex on cell leakage, the cytoplasmic contents of the cell were tested after exposure to the test compound. Cytoplasmic contents: potassium ions (K^+^), DNA, and proteins were selected as target molecules assayed for according to standard procedures. A cell concentration of 0.1 OD_600_ of *A. baumannii* was freshly inoculated in 50 mL sterile nutrient broth and grown overnight at 37 °C. Cells were harvested from the medium by centrifuging at 7500 rpm for 12 min. Cell pellets were separately re-suspended in 50 mL sterile saline solutions containing different concentrations (starting from the MIC) of DTC complex, Gen (at MIC) and DMSO controls, and incubated for 24 h at 37 °C. Samples of the supernatant were then obtained by separating cell pellets using centrifugation. Extracellular DNA leakage into the supernatant was quantified using a nanodrop spectrophotometer (ThermoFisher) by analyzing 1 µL of the sample and the concentration based on sample volume subsequently calculated. Proteins leaked into the supernatant were quantified using the Bradford assay technique^17^, while a flame photometer (Systronics) was used to assay for the presence of potassium ions in the supernatant^18^.

### Physiological effect of different concentrations of test DTC-Zn on planktonic cells of ***A. baumannii:*** ATP synthase assay

To further determine the metabolic effects of DTC-Zn complex on *A. baumannii*, biosynthesis of adenosine triphosphate (ATP) synthase was determined in treated isolates. Specifically, 120 mL of cell broths with 0.1 (OD 600) inocula were used. Cell pellets after resuspension in sterile saline with different ZnL1 concentrations were similarly incubated (as prepared above). The pellets were then harvested by centrifugation at 10,000 rpm for 12 min at 4 °C. To determine ATP synthase, cell membranes were extracted according to previously published protocols (Dadi 2010). Pellets were resuspended in 5 mL volume of (1 M) TES with 4.29 g Mg(CH_3_CO_2_)2·4H_2_O, 85.5 g sucrose, 0.0951 g EGTA and 5 g aminocaproic acid. The pH was adjusted at 6.5 with 1 M NaOH. Cells were stored at − 20 °C overnight, and afterwards were thawed, treated with DNAse and French-pressed through a cell fractionator at 2000 psi. Cell debris was centrifuged at 10,000 rpm for 30 min. Supernatants were collected and re-centrifuged at higher speed of 50,000 rpm for 100 min. The membranes were collected as pellets and stored at − 20 °C in a resuspension of 50 mM Tris Sulphate (pH 8) overnight. Cell membrane bound ATP synthase were assayed by mixing the cell pellets with 2 mL medium containing 100 mM KCl, 2.5 mM MgCl_2_, 2.5 mM HEPES, 10% glycerol, 2.5 mM K_2_HPO_4_, 650 µM ADP, and 50 µM Phenol Red which was added to the cell membrane pellets^19^. The assay reaction was started by addition of 3 mM disodium succinate, left to stand for 10 min and stopped by adding 3% sodium dodecyl sulphate. Change in colour of the phenol red indicator within the medium was as a result of pH changes resulting from the synthesis of ATP from ADP in the medium. The ATP synthase quantity was determined by spectrophotometric measurements of absorbance at 557 nm. The assay medium served as standard blank. ATP synthase was calculated using the formula:

$$ {\text{ATP synthase activity}}\left( {\frac{{\frac{{\upmu {\text{moL}}}}{{{\text{min}}}}}}{{{\text{mL}}}}} \right) = \frac{{{\text{Average sample OD}} - {\text{Average standard OD}}}}{{{\text{Quantity of sample }}\left( {{\text{mL}}} \right) \times {\text{Time }}({\text{min}})}} $$

### Spectroscopic analyses of treated and untreated cells

Cell pellets from earlier conducted cell leakage analyses were obtained by centrifugation and subjected to Fourier transform infrared (FTIR) spectroscopic analyses. Cells *treated with DTC-Zn (at MIC)* were compared with Gen treated cells (at MIC) and untreated cells. FTIR spectra were obtained between 400 and 4000 cm^−1^ using a Nicolet Impact 410 FTIR spectrometer at a resolution of 2 cm^−1^.

### Biofilm assays

Effects of antimicrobial treatments on forming biofilms (biofilm inhibition) and on pre-formed biofilms (biofilm removal) were carried out using the crystal violet assay technique^20^. Microtiter assays using a 48-well plate were carried out. Freshly prepared *A. baumannii* cells (0.5 OD_600_) were grown in nutrient broth. For biofilm inhibition, the cells were treated with varying concentrations of test compound ranging from 1 × MIC to 20 × MIC values. After introduction of compounds and inoculation, cells were incubated for 24 h at 37 °C. Gen at 1 × MIC and 10 × MIC were also used, and wells with only DMSO added without the test compounds served as the control. After incubation, media and planktonic cells were removed and discarded, while the wells were washed twice with sterile saline water. Biofilms were fixed by adding 99% methanol and allowing to stand for 10–15 min. Afterwards, wells were washed twice with sterile saline water, and air-dried, and the attached biomass were stained with 0.5% crystal violet solution for 20 min. Excess crystal violet was subsequently removed. The wells were gently washed and air dried. Biomass bound-crystal violet was solubilized by addition of 33% acetic acid and absorbance was read at 570 nm. For biofilm removal, cells were pre-cultured for 24 h after which the media with planktonic cells were discarded and the test compounds added before further incubation for 24 h. Percentages were used to express levels of biofilm inhibition or removal. The percentage of biofilm inhibited in comparison with the untreated control biofilms were calculated as reported in our previous work^21^. Triplicate experiments were conducted to obtain independent biological replicates.

### Electroanalysis

To determine the electrochemical response of *A. baumannii* cells to DTC-Zn treatments, inoculum of 0.5 OD_600_ was cultured overnight and inoculated into nutrient broth in EC. The SPE were connected to a computer-controlled multichannel potentiostat (Bio-Logic, France). SPEs were twice surface sterilized in 70% v/v ethanol, washed thrice in sterile deionized water, and air dried under aseptic conditions. Set-ups were made with DTC-Zn complex (at MIC) and Gen (at MIC) added. Experimental runs were carried out with 50 µM 2-HNQ added as mediator. Differential pulse voltammetry (DPV), and chronoamperometry (CA) were carried out on the different experimental iterations. DPV analyses were conducted at 0 h, and at the end of the CA, with scan rate of 5 mV s^−1^. Subsequently, the potential of the working electrode was set at 0.4 V vs*.* Ag, and the current output was recorded every minute for 24–48 h. The electrochemical cells were maintained at a temperature of 37 °C.

Electroanalyses of effects of DTC-Zn complex on pre-formed biofilm was carried out by first running the experimental set-ups inoculated with cells without the addition of test compounds till a stable current generation was observed (18–24 h). DTC-Zn and Gen control were added after stable current was observed by aseptic introduction using a sterile syringe. Current generated was monitored using CA, and electrical charge (mC) produced by each test condition was determined.

### Microscopic analyses

After electrochemical analyses, representative SPEs from the treated and untreated control set-ups were recovered and analysed for biofilm morphology and distribution using microscopic techniques. Electron microscopy was carried out using a scanning electron microscope (crossbeam 540; Carl Zeiss, Germany). Further microscopic analyses were carried out using a confocal laser scanning microscope (CLSM 780; Carl Zeiss, Germany). Biofilms of *A. baumannii* formed on the surface of the working electrode were targeted. After the removal of SPEs from EC set-ups, electrodes were gently washed in phosphate buffer solution (pH 7.3), and then treated with absolute ethanol for dehydration before fixation in formaldehyde (4% v/v). SPEs were finally dried at room temperature. Prior to analyses, samples for SEM analyses were coated with gold for 120 s using a Turbomolecular Pumped Coater Q150T (Quorum Technologies, UK). A 20 × objective was used in acquiring CLSM (Plan-Apochromat; NA = 0.8) and 2048 × 2048 resolutions (424.89 × 424.89 μm frame size with a pixel dwell time of 1.6 μs). Prior to CLSM analyses, biofilms on electrode were stained with 4′,6-diamidino-2-phenylindole (DAPI; Sigma-Aldrich) according to manufacturer’s directions. CLSM analyses involved the use of a 561-laser line, and autofluorescence was collected in the epi-illuminated geometry by the objective between 585 and 734 nm. Image analysis was done using Imaris imaging software.

## Results and discussion

### Preliminary screening, bacterial viability tests and MIC determination

Results from preliminary screening using agar diffusion experiments showed that ZnL1 and ZnL2 complexes had antibacterial activities against *A. baumannii* (Figure S1). However, *A. baumannii* was resistant to other test compounds screened. Interestingly, *A. baumannii* was resistant to the L1 ligand but was slightly susceptible to L2 when used alone. This implies that the bioactivity of ZnL1 and ZnL2 complexes is due to the Zn metal component of the complexes. ZnL1 was however more inhibitory than ZnL2 judging by the measured sizes of the ZOI (Table S1). Therefore, ZnL1 was used for further antibacterial experiments. However, the ZOI of the control drug (Gen 10 mM) was larger than the ZOI of ZnL1 at 10 mM.

The differences in activity can be attributed to the chemical structures of the ligands and the nature of their Zn complexes. L2, which has a phenyl and a piperazyl group, proved more potent than L1, which comprised a methyl and a phenyl group. However, the Zn complex formed with L1 was more effective than that formed with L2. The organic components of the DTC ligands have been identified as potent antibacterial and this can make the difference in activity between one DTC and another; however, this depends on their structure, orientation, molecular weight, and compatibility with target site within the bacterial cell^8^. It has also been reported that bacterial susceptibility to DTCs is enhanced when metals are coordinated with the DTC ligands, thereby forming ligand–metal complexes^22^. Metallic Zinc has been reported as a strong antimicrobial^10^. This makes it a viable candidate for designing safe and effective drug when complexed with DTCs. All the DTCs and DTC-Zn complexes used in this preliminary antibacterial screening were originally designed as anticancer agents. Repurposing ZnL1 was therefore targeted in a bid to complement its anticancer activity.

**Figure 1:**
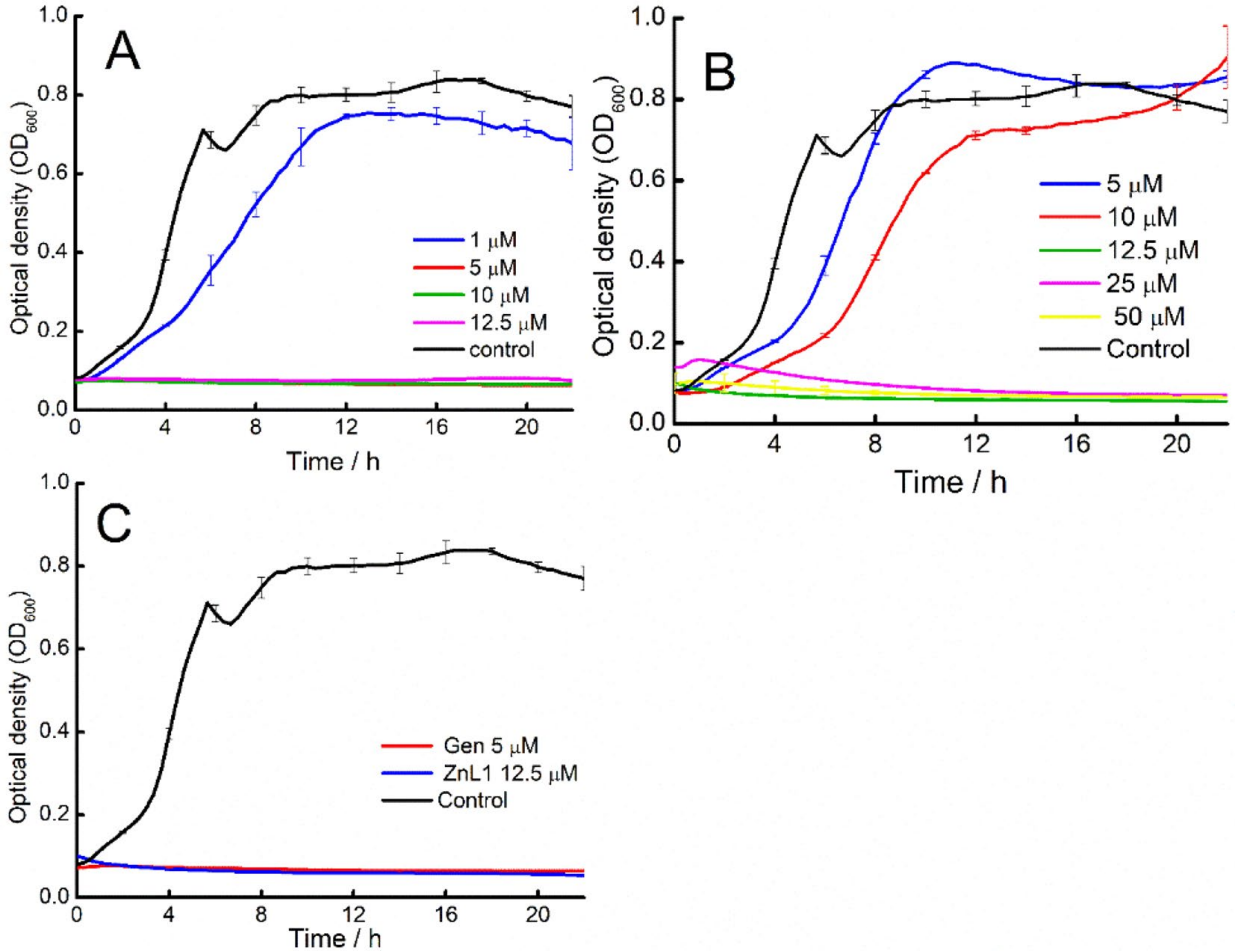
Effect of different concentrations of tested antibacterial compounds on growth of *A. baumannii*: (**A**) Gen; (**B**) ZnL1; (**C**) MIC of Gen and ZnL1.

A concentration-dependent antibacterial activity against planktonic cells was observed with ZnL1, in the range 5–50 µM. From the microdilution experiments the MICs for ZnL1 and Gen against *A. baumannii* were 12.5 µM and 5 µM, respectively (Fig. 1). Previous reports of the use of Zn alone as an antimicrobial agent were carried out using ZnSO_4,_ and the data obtained suggests that Zn concentrations able to induce stress on *A. baumannii* were at 400 µM, however full delay of bacterial growth occurred only with concentrations of Zn at 600, 800 and 1000 µM. This clearly puts the role of antimicrobial activity on the DTC ligands in this case; as our experiments here have shown an activity of ZnL1 at 12.5 µM, a factor of over 30 times efficacy than what is achieved with Zn alone. The ligands and ligand-complexes were dissolved in DMSO. DMSO treated cells served as the control as DMSO did not alter the growth curve. Furthermore, the lag phase increased even at sub/inhibitory concentrations (5 and 10 µM) of ZnL1. The low MIC value for ZnL1, which is not much lower than that of Gen, indicates that ZnL1 has good potentials as an antibacterial agent.

Cell viability experiments in previous works with DTC against a *Mycobacterium marinum* strain have also shown a concentration-dependent effect of DTCs on bacterial growth with an MIC of 75 µM^23^. Tests on *A. baumannii* using DTCs have been reported, however this is the first report on the antibacterial activity of the newly synthesized phenylpiperazyldithiocarbamate-Zn complex against *A. baumannii*. In many cases of metal-DTC complexes, the antibacterial activities on bacterial species like *Escherichia coli*, *Pseudomonas aeruginosa*, *Salmonella Typhimurium* and fungi such as *Candida albicans* and *Aspergillus niger* are enhanced when metal components such as Cobalt, Iron, Nickel, Copper, and Manganese are added^8,22^. The application of ZnCl_2_ and pyrrolidine dithiocarbamate, (CH_2_)_4_NCS_2_^(−)^ against *Staphylococcus aureus* and *Escherichia coli* showed an increased effect than DTC alone^8^. Comparative studies investigated the effect of different metals, like zinc, cobalt, copper and manganese on the antibacterial potency of *N*-methyl-*N*-phenyldithiocarbamate, when stable complexes of the metals were formed with the DTC. The highest antimicrobial activity was recorded for the Zn complexes against *Streptococcus pneumoniae, Staphylococcus aureus, E. coli* and the fungus *Aspergillus niger*^22^.

### Physiological effects of different concentrations of test DTC-Zn on planktonic cells of *A. baumannii*

**Figure 2:**
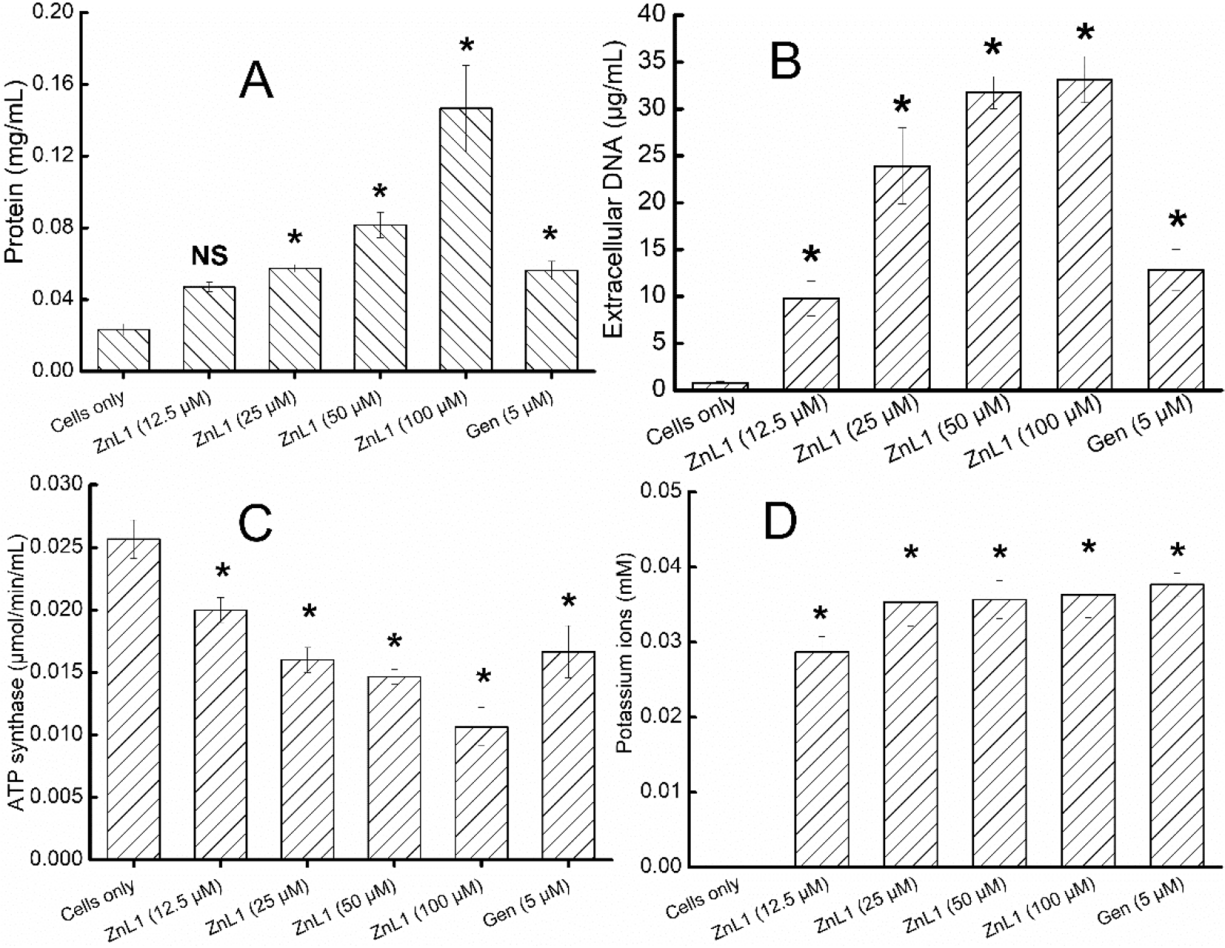
Effect of different concentrations of tested drugs on selected cell physiological function indices to determine cell leakage and ATP synthase loss; (**A**) intracellular protein leakage; (**B**) DNA leakage; (**C**) ATP synthase loss; (**D**) potassium ion leakage. Error bars represent standard deviation (SD) of at least three independent replicates. *NS* not significant, ∗  significant difference between treated cells in comparison to the untreated cells (*p* < 0.05) according to ANOVA followed by Tukey’s test (Tables S2 (A, B C and D)).

To further elucidate the specific effects of ZnL1 on *A. baumannii*, we investigated the roles played by ZnL1 in inducing cell leakage, and loss of ATP synthase activity. For cell leakage assays, cytoplasmic contents, potassium ions (K^+^), DNA, and proteins were assayed for in cell-free extracts, after antibacterial treatments. Results showed that there was concentration-dependent leakage of intracellular components from the cells (Fig. 2), in the range 12.5–100 µM (1–8 × MIC). The effect of ZnL1 on cell leakage suggests that ZnL1interferes with cell envelope components of *A. baumannii*. The effect observed for ZnL1 at 1 × MIC (12.5 µM) were similar to those for Gen at 1 × MIC (5 µM), with the exception of potassium ion concentration, which was significantly higher for Gen than for ZnL1. However, Gen is an aminoglycoside that targets the nucleic materials of bacterial cells. Therefore, its involvement in cell wall breakage and leakage of cell components as shown here may be indirect. Gen is reported to be associated with the cell death process because it interferes with protein synthesis and caused internal cellular damage, subsequently causing cell rupturing^24^, which then lead to the release of intracellular components of the cell into the medium.

The leakage of intracellular proteins into the medium following exposure to ZnL1 is particularly interesting as it signals a comprehensive loss of functionality of the *A. baumannii* cells as the proteins are major tools of cellular functions^25^. DNA leakage from cells was also concentration dependent and reach a maximum of ~ 32 µg mL^−1^, which is quite significant owing to the fact that DNA are primarily conserved intracellular components of cells and its leakage into the medium signifies cell rupturing and cell wall degradation. While extracellular DNA is commonly observed in biofilm and contributes to its physiology, rapid loss of DNA in planktonic cells following exposure to antimicrobials implies loss of cell wall integrity^26^. ZnL1 also led to the seepage of potassium ions from *A. baumannii* cells. Potassium are important physiological elements that are highly essential in maintenance of cellular respiration and ionic balance as well as membrane integrity^27^. Potassium ions lost from the cells reached values > 0.03 mM. Cellular loss of potassium, however, was not concentration dependent and similar following exposure to ZnL1 and Gen.

The effect of ZnL1 on cell viability was also confirmed by the assay of ATP synthase, which was also concentration dependent. ATP synthases are membrane bound enzymes. This implies that in many cases of cell rupturing, the enzymes will be attached more to the resulting cell pellets and cellular debris. Results show that there was reduction in ATP synthase detected. This could be attributed to the degradation of cell pellets after the treatments with test compounds (ZnL1 and Gen). Reduction in cell pellets can be directly associated with reduction in biomass caused by the inhibitory influence of the compounds on planktonic cell growth. The reduced biomass led to a reduction in detectable presence of ATP synthase as shown by the ATP activity assay.

### Spectroscopic analyses of treated and untreated cells

**Figure 3:**
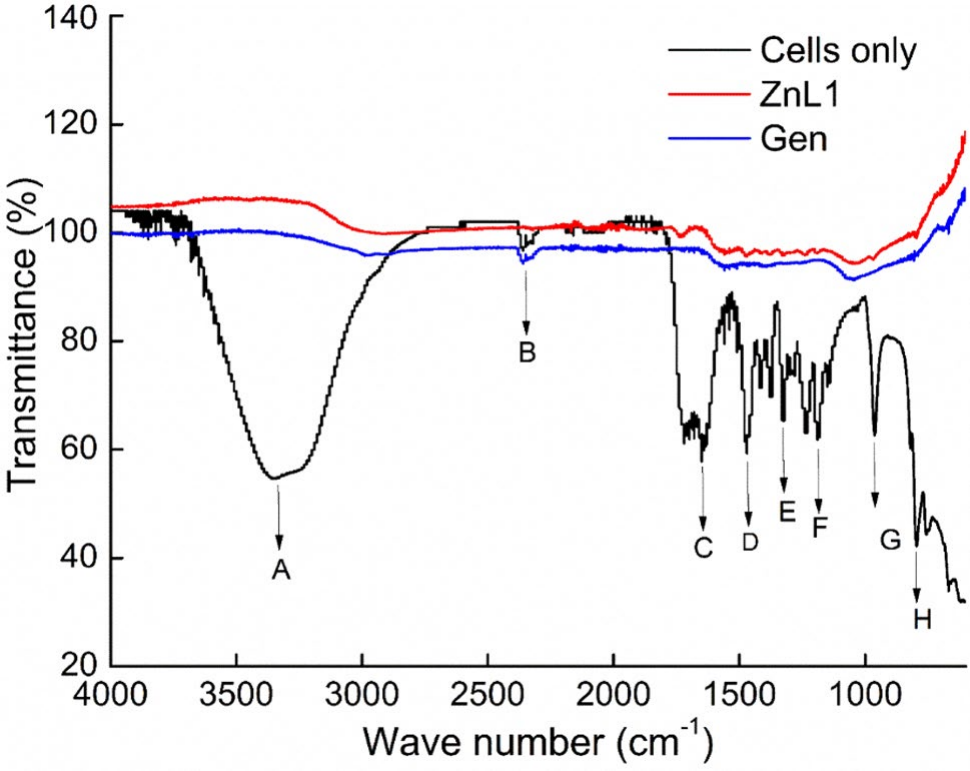
Representative FTIR spectra of cell pellets from *A. baumannii* treated with MIC of ZnL1 compared with MIC of Gen and untreated control.

To further confirm the effects of ZnL1 on the structure of the cell pellets obtained from the centrifuged broth, FTIR analyses were carried out after treatments with ZnL1 and Gen at 1 × MIC on the cells. All unbound organic compounds including media and drugs were washed off from the samples before analyses, leaving only cellular components. The spectra of the treated cells were markedly different from those of the untreated cells. Healthy, untreated cells of *A. baumannii* showed representative peaks indicating organic components of viable bacterial cells. The following active chemical groups of A; O–H and N–H protein amide (3329.25 cm^−1^), B; O=C=O isocyanate (2352 cm^−1^), C; Amide I protein (1639.60 cm-1), D; Amide II protein (1466.25 cm^−1^), E and F; C–O, C–C, C–OH, C–O–H, C–O–C polysaccharides (1174.4–1300 cm^−1^), G; O–P=O symmetric stretching vibrations (960 cm^−1^), and H; C=O (805 cm^−1^) were all prominent peaks detected within the range from 400 to 4000 cm^−1^. These peaks were detected in cell pellets of untreated control, while a drastic reduction in all prominent peaks was observed in all tests samples of ZnL1 (Fig. 3).

The absence of the peaks of the functional chemical groups within the treated samples is indicative of degradation of cellular components that constitute the cell pellets that were used for analyses. FTIR analyses of the untreated cell pellets had chemical groups associated with cellular proteins, polysaccharides, lipids, and other organic constituents. FTIR spectra of cellular components have been employed in studying slight or extensive alterations in cellular compositions affected by chemical and biochemical treatments. Microbiological analyses coupled with FTIR spectroscopy had in previous works^28^ also showed the links between cellular viability and the presence of similar functional groups as reported in our work. Cellular changes were evident when planktonic cells were utilized, however the case might have been different with biofilms. We therefore sought to investigate the response of biofilms of *A. baumannii* to treatment with ZnL1.

### Biofilm assay

**Figure 4:**
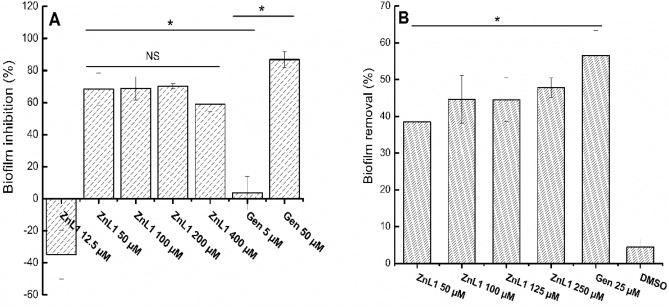
Effects of ZnL1 in comparison with Gen, on forming and preformed biofilms of *A. baumannii*. (**A**) Effects on forming biofilm–biofilm inhibition (**B**) Effects on pre-formed biofilm–biofilm removal. Error bars represent SD of at least three independent replicates. *NS* not significant, ∗   significant difference between marked experimental conditions (*p* < 0.05) according to ANOVA followed by Tukey’s test (Table S3 (A and B)).

Biofilms have been associated with the heightened level of drug resistance exhibited by many clinically important strains of *A. baumannii*^1^. Thus, it is necessary to assess the antibiofilm properties of ZnL1 to determine its suitability for real applications. Here, we focused on two standard biofilm testing methods: (a) inhibition of biofilm formation and (b) removal of pre-formed biofilms. Results showed that ZnL1 inhibits biofilm formation by 60% when applied at high concentration > 50 µM (4 × MIC). However, the biofilm inhibition effect did not change much at higher concentration, up to 32 × MIC. This may be due to low sensitivity of crystal violet in detecting biofilm inhibition. Similar trend was observed for Gen, in which a concentration as high as 10 × MIC is needed to observe a strong biofilm inhibition. These results are consistent with previous literature on biofilm inhibition (Fig. 4A). Biofilms have been proven to be tougher than planktonic cells systems due to their high impermeability to external antimicrobial agents^29,30^. Higher dose of compounds when applied, can make the biofilm more susceptible and eventually yields to the antimicrobial treatments. At 1 × MIC, both ZnL1 and Gen appeared to cause no decrease in biofilm formation, in fact there was more biofilm formed after this concentration was applied. This could be as a result of the artifacts from dead cells that were detected alongside viable cells, as the crystal violet binding assay is not entirely specific for viable cells. It can also be as a result of insufficient antimicrobial concentrations, as sub-toxic antimicrobial treatments can stimulate mild biofilm formation^31–34^.

Biofilm removal assessed for pre-formed biofilms showed that efficient biofilm removal for ZnL1 was achieved when up to 100 µM (8 × MIC) values were applied (Fig. 4B). Approximately above 45% biofilm removal was observed with the test compound (ZnL1), and similarly like in biofilm inhibition (Table S2), there was no significant difference between the values of biofilm removal achieved at 100, 200, 250 µM (× 8, × 16, × 20 MIC values) (Table S3) pointing to the fact that higher concentrations add little effects on biofilm removal efficiency of ZnL1. This is due to the fact that biofilm inactivation and removal are different. As inactivation is purely a chemical process, biofilm removal is chemo-mechanic, requiring fluid flow for dislodgement of biofilms. In our experiments, we used static systems. This could account for the results obtained that showed no obvious effect of increased concentrations of ZnL1 on biofilm removal. Gen at 100 µM (20 × MIC) performed better that ZnL1, as biofilm removal efficiency of above 55% were obtained in the experiments. ZnL1 has shown good promise in antibacterial efficacy so far. The instances of higher antimicrobial effects observed with Gen as a conventional antibiotic however are quite understandable as this is usually the case with newly designed chemotherapeutic agents. Further optimization of the chemical structure of the compound will be necessary for improved efficacy especially against biofilms. It could be added that their efficiency in biofilm formation could be tied to the modes of activity of the two compounds. So far, we hypothesise that ZnL1 targets the outer cell envelope while we understand that Gen targets protein synthesis^25^. Proteins are a major part of biofilms and so far, we have not ascertained if ZnL1 is proteolytic in nature, however the proven protein inhibition mechanism of Gen which involves the targeting of the protein translation in bacteria makes it a potent broad spectrum antibiotic and potentially stronger anti-biofilm agent than ZnL1.

To further determine the time-based dynamics in antibiofilm efficiency of the ZnL1 in comparison with Gen, we sought to electroanalytically evaluate their antibiofilm potentials by monitoring metabolism of *A. baumannii* biofilms after treatments with test drugs, using extracellular electron transfer as the index^21,35,36^. Effects on gradually forming biofilms as well as pre-formed biofilms were assessed in a time-dependent fashion.

### Electroanalyses of effects of DTC-Zn complex on biofilm of *A. baumannii*

**Figure 5:**
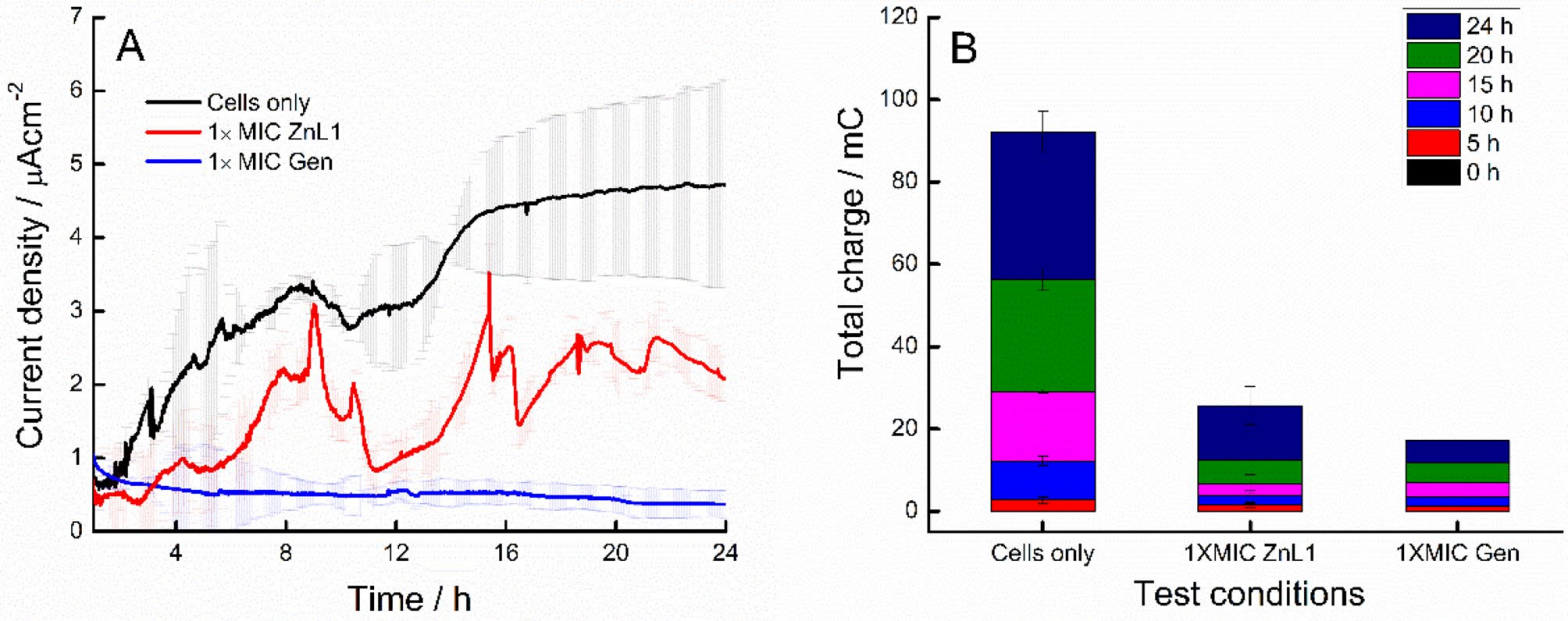
(**A**) Chronoamperometry analyses showing current trend of 24 h growth of forming *A. baumannii* biofilms with and without antimicrobial treatments; 5 µM Gen (1 × MIC) and 12.5 µM ZnL1 (1 × MIC) were used in comparison with untreated control. (**B**) Total charge (mC) produced by *A. baumannii* biofilms between 0 and 24 h for the different conditions.

Figure 5 shows the chronoamperometric plots of treated and untreated biofilms forming on SPEs. The current output increased with time for untreated cells, until the maximum current (> 5.56 µA cm^−2^), which was reached at 24 h. On the other hand, the current output was much less regular in presence 1 × MIc ZnL1 and the maximum current produced was < 3.17 µA cm^−2^. Finally, the current declined slowly in presence of 1 × MIC Gen, with a maximum current output of 0.79 µA cm^−2^. Thus, ZnL1 was less effective than Gen in decreasing the extracellular redox flow. The irregular profile of current output in cells exposed to ZnL1 has been observed in other bioelectrochemical studies of antimicrobial agents^21^.

**Figure 6:**
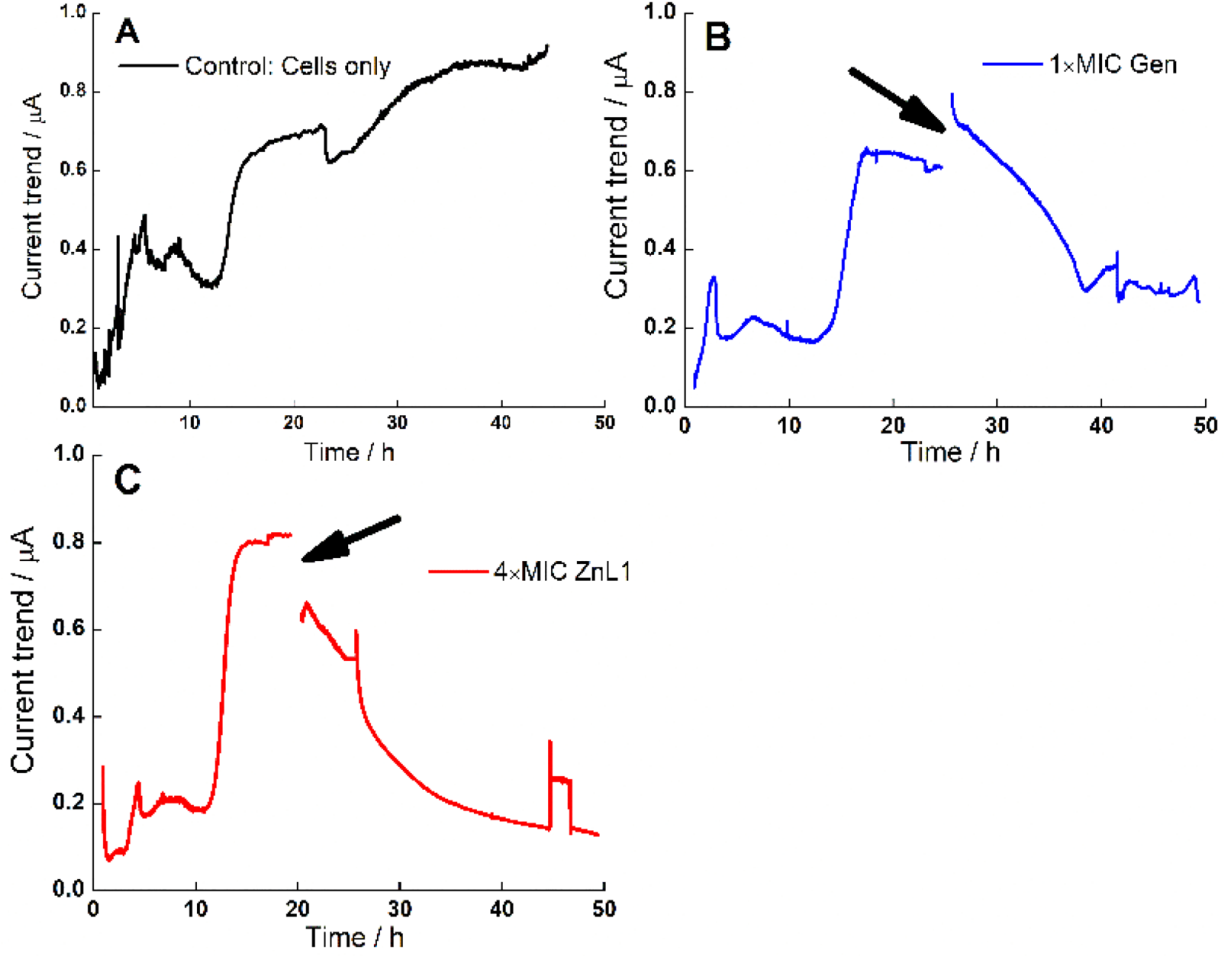
Representative chronoamperometric plots of current generated by pre-formed biofilms of *A. baumannii* after addition of ZnL1 (4 × MIC) in comparison with addition of Gen (1 × MIC) and untreated controls (**A**) untreated control cells (black trace); (**B**) cells treated with 1 × MIC Gen (blue trace) and (**C**) Cells treated with ZnL1 (4 × MIC) (red trace). Point of addition of the drug is marked with an arrow (black).

The effects of ZnL1 and Gen at 1 × MIC on the electrical current output of pre-formed biofilms was also measured (Fig. 6). ZnL1 was effective in reducing current produced when applied at 50 µM (4 × MIC) in comparison with similar antibacterial impacts as observed with Gen at 5 µM (1 × MIC). A steady electrical current was achieved for pre-formed biofilms at between 15 and 24 h of growth, and that signified the point of addition of test antimicrobials. Steady declines in current generated after the addition of antimicrobials was observed with both ZnL1 and Gen treatments. Biofilm formation has been extensively associated with cellular electrical current output, and in our previous work, we had addressed chronoamperometric approaches to determining inhibitory effects of antibiofilm agents on *A. baumannii* biofilms^21^. It has been observed that though electrical charge production in biofilms is as a result of cellular respiration, there still is a direct correlation between biofilm biomass formed and the totality of cellular respiration occurring within the biofilm structures^37^. This invariably implies that the increase or decrease in biomass attributed to altered respiration can be a point of detection as earlier proven. This forms an analytical tool for rapid detection and studies of drug activity within bacterial systems. Biomechanisms of these drugs against bacterial respiration in biofilms can also be further elucidated using bioelectrochemical approaches.

**Figure 7:**
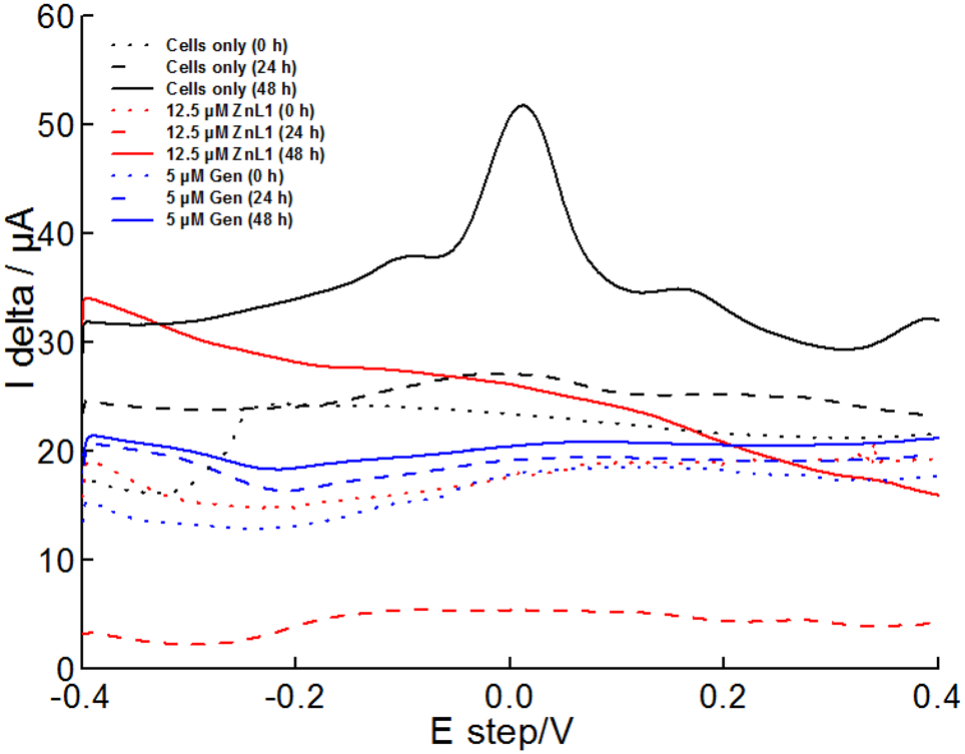
Electrochemical responses of *A. baumannii* cells treated with ZnL1 and Gen at MIC. Differential pulse voltammetry analyses of treated and untreated *A. baumannii* biofilms with up to 48 h exposure.

The electrochemical signature of treated and untreated biofilms grown on screen-printed electrodes was determined through differential pulse voltammetry (DPV). Within biofilm, different species such as redox enzymes and extracellular material could elicit varying degrees of electrical current/charge at varied potentials^38^. DPV analyses were carried out immediately after inoculum and every 24 h until the end of the experiments (Fig. 7). Biofilm accumulation is linked with production of defined electrochemical peaks, due to the accumulation of redox-active species^39^. It was observed that full electrochemical signatures were achieved with 48 h old biofilms (Fig. 7).

At 48 h, clearly distinct voltage peaks were observed at − 0.1 V, 0.02 V, and 0.19 V. The highest peak was at approximately 0.02 V. Bioelectrochemical analyses of electroactive and weakly electroactive bacteria shows that biofilms from such bacteria can elicit electrical charge due to electroactive cellular biomass or as a result extracellularly released redox components^38^. In this work, 2-HNQ was used as a redox mediator to assist electron harvesting from weakly electroactive *A. baumannii* biofilms. From reports using 2-HNQ as redox mediator in electroanalyses of weak electricigens, it could be determined that the positive peak at 0.02 V is possibly attributed to cellular biomass attached to the SPEs and not as a result of extracellularly secreted redox compounds^40^. The other smaller peaks at − 0.1 V and 0.19 V are not fully elucidated here, however, they are suspected to be attributed to other redox active species within the biofilm mass like cytochromes and flavins^41^. With the introduction of the antimicrobial agents (ZnL1 and Gen), there was a complete loss of the voltage peaks even as the biofilm analyses progressed for up to 48 h. Under antimicrobial treatments, there were changes in current (I delta) generated ranging between 2 and 30 µA for the different sequential times (0, 24 and 48 h) assessed under the influence of ZnL1 and Gen treatments, however this did not show definite voltage peaks as observed at 48 h with the untreated control. This signified that ZnL1 and Gen both altered biofilm respiration by changing redox processes thereby hindering the complete physiological functioning of the biofilms.

DPV experiments to determine the electrochemical signature of *A. baumannii* biofilms exposed to ZnL1 at sub-inhibitory concentrations (Figure S3) showed that in presence of 0.1 µM ZnL1, the DPV peak increase with time, showing a main peak at 50–60 mV vs Ag. At 1 µM ZnL1, the DPV peak is much smaller and increases both in potential and height as the biofilm grows. At higher concentration (5 and 10 µM), no evident peak is observed. These results are consistent with charge output and biochemical experiments. However, the concentration of ZnL1 at which the DPV peak is not observed is approximately 5 times smaller than the MIC, indicating that sub-toxic concentrations of ZnL1 affect the extracellular respiration and electrochemical signature of *A. baumannii.*

We hypothesize here that the redox altering properties of ZnL1 against *A. baumannii* biofilms can also be associated with altering membrane metalloproteins present in *A. baumannii*. Previous reports by Hassan and colleagues had shown that exerting metal stress on *A. baumannii* by exposing cells to zinc concentrations as high as 400–1000 µM could negatively impact on the viability and growth of *A. baumannii* cells as oxidative shock could be induced in the cells^11^. It is important to note however that the concentrations used in our work were much smaller and far below the concentrations linked with potential oxidative stress. Therefore, another plausible explanation for the mechanism of ZnL1 activity is needed. Zinc has been reported to be a very bio-friendly metal in many living systems especially at lower concentrations; it therefore makes sense that ZnL1 could be easily assimilated into the bacterial cell systems. Within the bacterial system, Zinc could dissociate from the coordination complex with the L1 DTC ligand, freeing the ligand to interact with available metals within metalloproteins present within the membrane of *A. baumannii*. This interaction could chelate more metals, thereby ridding the membrane of available metals to balance its ionic flow and integrity. This could therefore spiral into osmotic shock, cell turgidity and rupturing of cell membrane.

### Microscopic analyses

**Figure 8:**
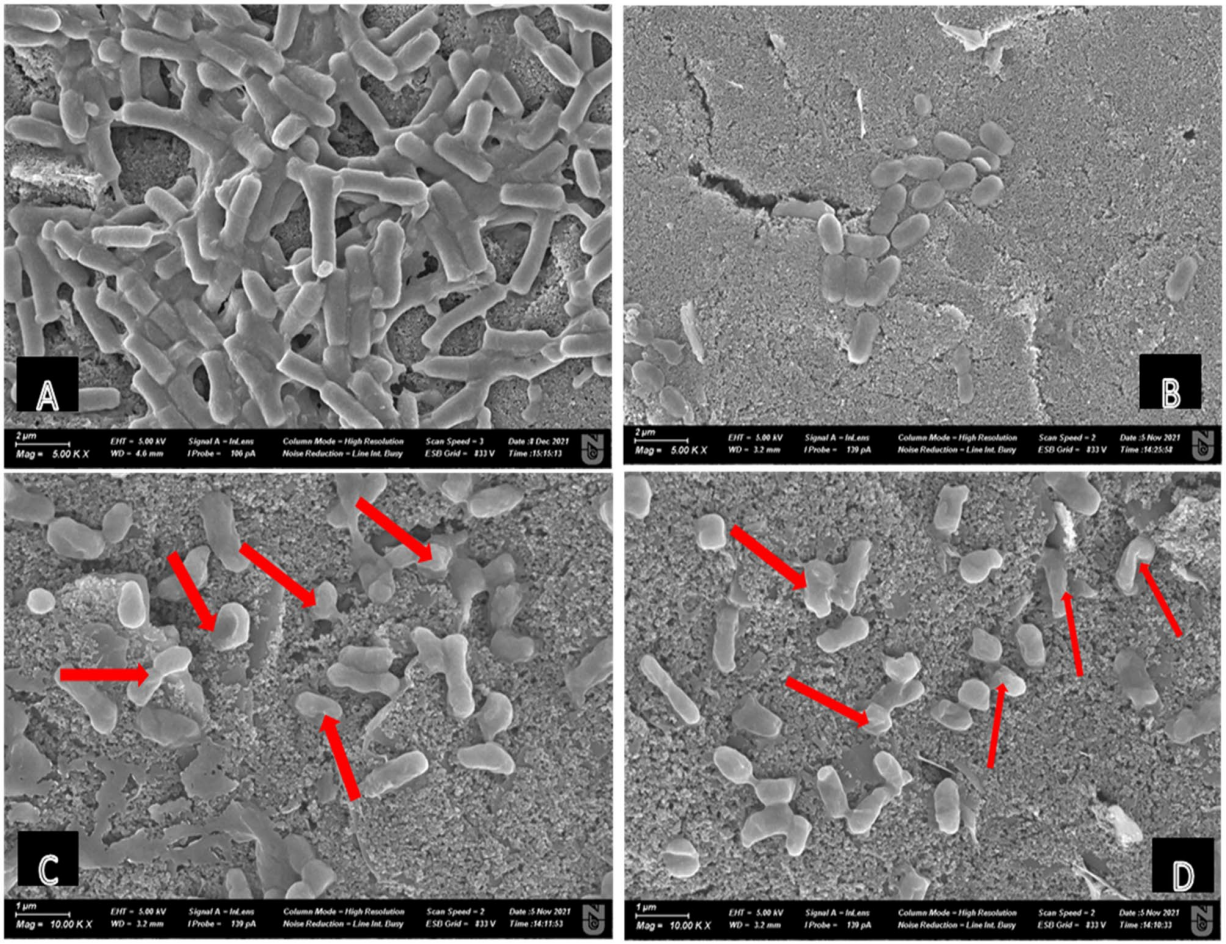
Representative scanning electron micrographs of *A. baumannii* on the working electrode area only (**A**) cells not treated with any of the tested compounds—Control; (**B**) cells treated with Gen; (**C**,**D**) cells treated with ZnL1. Red arrows showing point of ell rupture and shrinkage.

**Figure 9:**
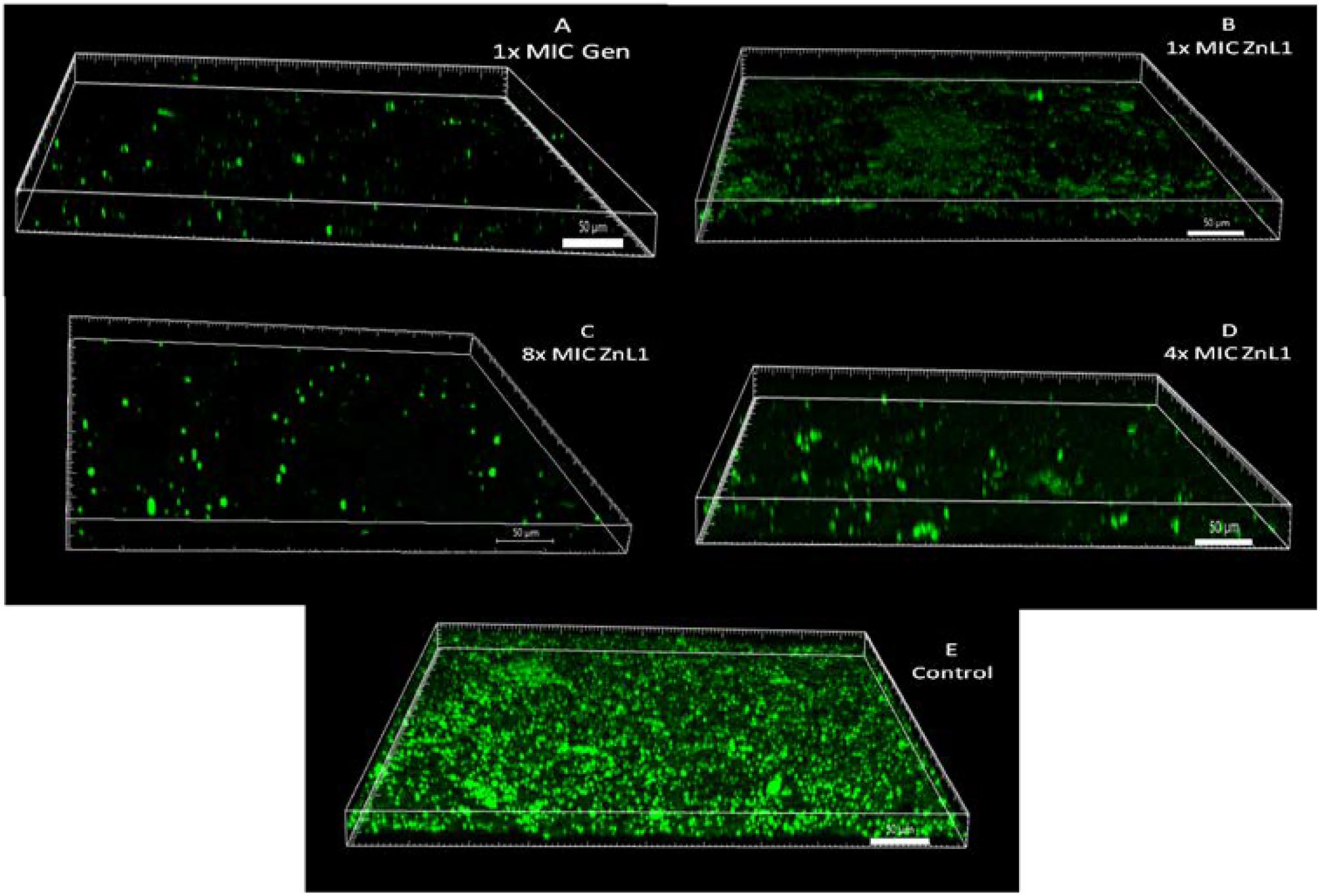
Representative CLSM images of *A. baumannii* analysed on the working electrode (**A**) Cells exposed to 1 × MIC, Gen (**B**) Cells exposed to 1 × MIC, ZnL1 (**C**) Cells exposed to 8 × MIC, ZnL1 (**D**) Cells exposed to 4 × MIC, ZnL1 (**E**) Untreated cells—control.

To ascertain the potentials for inducing cell rupturing effects on *A. baumannii* cells, we employed microscopic techniques to investigate cellular morphology after treatments of the cells. Images from scanning electron microscopy (Fig. 8) and confocal laser scanning microscopy (Fig. 9) revealed that cell rupturing and biofilm reduction effects were respectively evident. There was decline in viable cells as visibly wrinkled cells were shown in electron micrographs after Znl1 treatments, quite contrasting with the luxuriant, healthy cell forms in untreated controls. Cell rupturing was also evident as the cells shrunk in size after ZnL1 treatment, depicting flaccid structures that had lost intracellular contents. Fluid loss, flaccidity, and cell rupturing are evident features of unhealthy cells that have been exposed to antimicrobial agents^21^. This was however visibly different from the way the cell structures appeared after Gen, determining that the possible cell rupturing process evident after ZnL1 may not exactly have been the same case for Gen treated cells. There was, however, drastic reduction of cell populations in both ZnL1 and Gen treated conditions.

Figure 7 showed CLSM images of biofilms captured after DAPI staining. Images revealed loss of cell biomass in comparison with the untreated controls. The observed influence of increased concentration showed that viable cells were affected in a concentration dependent manner when treated with ZnL1. This was however not exactly the case with earlier biofilm inhibition and removal assessments using ZnL1 as there were no significant differences in biofilm removal or inhibition among treatments with varied concentrations when crystal violet biding assay was used. Azeredo and colleagues had however stated that the crystal violet technique was not differential and could not selectively detect living cells during the assay^42^. In such case, both living and dead cells were captured during the CV assay. This could account for the difference in significance in biofilm removal/inhibition when tested under crystal violet technique and CLSM technique. The presence of viable cells herein determined the efficiency of ZnL1 at increasing concentrations. Gen at 1 × MIC however still showed to be more effective than ZnL1 at 1 × MIC.

Overall, the results show that the effect of ZnL1 as antibacterial agent against *A. baumannii* is due to both DTC ligands and the metal centers, in this case, zinc. The metal-DTC ligands due to the possibility of dissociation within the cell membrane can alter membrane ionic balance and cell respiration by reacting with metalloproteins. ZnL1 had previously been demonstrated to have antiproliferative cytotoxic activity against TK10, UACC62 and MC7 human cancer cell lines with a IC_50_ ranging from 14.28 to 22.74 µM^12^. It is not uncommon to observe that antitumoral compounds have some effect on bacterial pathogens^7,43^. However, experiments on animal models are needed to determine the exact range and efficacy of this compound, especially against biofilms.

## Conclusions

A dithiocarbamate-metal complex (ZnL1) previously synthesized between a dithiocarbamate ligand and zinc metal to yield *N*-methyl-1-phenyldithiocarbamato-S,S′ zinc(II) has been demonstrated to have activity against *A. baumannii* planktonic cells and biofilms. ZnL1 was previously designed as an anticancer agent with an IC_50_ for human cancer cell lines ranging from 14.28 to 22.74 µM. However, repurposing of ZnL1 showed antibacterial effect of ZnL1 on planktonic cells of *A. baumannii* at MIC (12.5 µM). This was comparable with the effects of Gen also at MIC (5 µM). Electroanalysis, physiological and microscopic assays of treated *A. baumannii* biofilms demonstrated efficacy of ZnL1 in inhibiting biofilm formation and removing pre-formed biofilms. Hypothetically, ZnL1 can alter metalloprotein functions within the cell membrane as a mechanism to induce reduction in cell respiration. This needs to examined and proved with further experiments to confirm this mechanism. Given its strong effect, ZnL1 could be used in formulation to target biofilms for in vivo applications.

## Supplementary Information

Supplementary Information.

## Data Availability

The datasets generated and/or analysed during the current study are available in the figshare repository (figshare.com; https://doi.org/10.6084/m9.figshare.19747621).

## References

[1] Vázquez-López R (2020). *Acinetobacter baumannii* resistance: A real challenge for clinicians. Antibiotics.

[2] Raut S (2020). Trend and characteristics of *Acinetobacter baumannii* infections in patients attending universal college of medical sciences, Bhairahawa, Western Nepal: A longitudinal study of 2018. Infect. Drug Resist..

[3] Morris FC, Dexter C, Kostoulias X, Uddin MI, Peleg AY (2019). The mechanisms of disease caused by *Acinetobacter baumannii*. Front. Microbiol..

[4] Antunes LCS, Imperi F, Carattoli A, Visca P (2011). Deciphering the multifactorial nature of *Acinetobacter baumannii* pathogenicity. PLoS One.

[5] Kyriakidis I, Vasileiou E, Pana ZD, Tragiannidis A (2021). *Acinetobacter baumannii* antibiotic resistance mechanisms. Pathogens.

[6] CDC. *Antibiotic resistance threats in the United States*. *Centers for Disease Control and Prevention* https://www.cdc.gov/drugresistance/biggest_threats.html (2019) 10.15620/cdc:82532.

[7] Odularu AT, Ajibade PA (2019). Dithiocarbamates: Challenges, control, and approaches to excellent yield, characterization, and their biological applications. Bioinorg. Chem. Appl..

[8] Yeo CI, Tiekink ERT, Chew J (2021). Insights into the antimicrobial potential of dithiocarbamate anions and metal-based species. Inorganics.

[9] Shinde SD, Sakla AP, Shankaraiah N (2020). An insight into medicinal attributes of dithiocarbamates: Bird’s eye view. Bioorg. Chem..

[10] Kambe T, Tsuji T, Hashimoto A, Itsumura N (2015). The physiological, biochemical, and molecular roles of zinc transporters in zinc homeostasis and metabolism. Physiol. Rev..

[11] Hassan KA (2017). Zinc stress induces copper depletion in *Acinetobacter baumannii*. BMC Microbiol..

[12] Andrew FP, Ajibade PA (2018). Synthesis, characterization and anticancer studies of bis-(N-methyl-1-phenyldithiocarbamato) Cu(II), Zn(II), and Pt(II) complexes: Single crystal X-ray structure of the copper complex. J. Coord. Chem..

[13] Andrew FP, Ajibade PA (2018). Synthesis, characterization and anticancer studies of bis(1-phenylpiperazine dithiocarbamato) Cu(II), Zn(II) and Pt(II) complexes: Crystal structures of 1-phenylpiperazine dithiocarbamato-S, S′ zinc(II) and Pt(II). J. Mol. Struct..

[14] Ajibade PA, Andrew FP, Botha NL, Solomane N (2020). Synthesis, crystal structures and anticancer studies of morpholinyldithiocarbamato Cu(II) and Zn(II) complexes. Molecules.

[15] Ajibade PA, Andrew FP, Fatokun AA, Oluwalana AE (2021). Synthesis, characterization and in vitro screening for anticancer potential of Mn(II), Co(II), Cu(II), Zn(II), and Pt(II) methoxyphenyl dithiocarbamato complexes. J. Mol. Struct..

[16] Yang CH, Su PW, Moi SH, Chuang LY (2019). Biofilm formation in *Acinetobacter baumannii*: Genotype–phenotype correlation. Molecules.

[17] Bradford MM (1976). A rapid and sensitive method for the quantitation microgram quantities of protein utilizing the principle of protein-dye binding. Anal. Biochem..

[18] Banerjee P, Prasad B (2020). Determination of concentration of total sodium and potassium in surface and ground water using a flame photometer. Appl. Water Sci..

[19] Feniouk BA, Suzuki T, Yoshida M (2007). Regulatory interplay between proton motive force, ADP, phosphate, and subunit ε in bacterial ATP synthase. J. Biol. Chem..

[20] Stepanovic S (2007). Quantification of biofilm in microtiter plates: Overview of testing conditions and practical recommendations for assessment of biofilm production by staphylococci. APMIS.

[21] Olaifa K, Ajunwa O, Marsili E (2022). Electroanalytic evaluation of antagonistic effect of azole fungicides on *Acinetobacter baumannii* biofilms. Electrochim. Acta.

[22] Ekennia AC, Onwudiwe DC, Olasunkanmi LO, Osowole AA, Ebenso EE (2015). Synthesis, DFT calculation, and antimicrobial studies of novel Zn(II), Co(II), Cu(II), and Mn(II) heteroleptic complexes containing benzoylacetone and dithiocarbamate. Bioinorg. Chem. Appl..

[23] Aspatwar A (2017). β-CA-specific inhibitor dithiocarbamate Fc14–584B: A novel antimycobacterial agent with potential to treat drug-resistant tuberculosis. J. Enzyme Inhib. Med. Chem..

[24] Liu JY (2016). In vitro activity of aminoglycosides against clinical isolates of *Acinetobacter baumannii* complex and other nonfermentative Gram-negative bacilli causing healthcare-associated bloodstream infections in Taiwan. J. Microbiol. Immunol. Infect..

[25] Kohanski MA, Dwyer DJ, Collins JJ (2010). How antibiotics kill bacteria: From targets to networks. Nat. Rev. Microbiol..

[26] Hartmann M (2010). Damage of the bacterial cell envelope by antimicrobial peptides gramicidin S and PGLa as revealed by transmission and scanning electron microscopy. Antimicrob. Agents Chemother..

[27] Yao C, Li X, Bi W, Jiang C (2014). Relationship between membrane damage, leakage of intracellular compounds, and inactivation of Escherichia coli treated by pressurized CO2. J. Basic Microbiol..

[28] Kamnev AA (2021). Fourier transform infrared (FTIR) spectroscopic analyses of microbiological samples and biogenic selenium nanoparticles of microbial origin: Sample preparation effects. Molecules.

[29] Abebe GM (2020). The role of bacterial biofilm in antibiotic resistance and food contamination. Int. J. Microbiol..

[30] Shenkutie AM, Yao MZ, Siu GKH, Wong BKC, Leung PHM (2020). Biofilm-induced antibiotic resistance in clinical *Acinetobacter baumannii* isolates. Antibiotics.

[31] Penesyan A, Paulsen IT, Gillings MR, Kjelleberg S, Manefield MJ (2020). Secondary effects of antibiotics on microbial biofilms. Front. Microbiol..

[32] Kaplan JB (2012). Low levels of β-Lactam antibiotics induce extracellular DNA release and biofilm formation in Staphylococcus aureus. MBio.

[33] Teh AHT, Lee SM, Dykes GA (2019). Growth in the presence of specific antibiotics induces biofilm formation by a *Campylobacter jejuni* strain sensitive to them but not in resistant strains. J. Glob. Antimicrob. Resist..

[34] VinodKumar K (2018). Can subminimal inhibitory concentrations of antibiotics induce the formation of biofilm in Leptospira?. Microb. Drug Resist..

[35] Naradasu D, Guionet A, Miran W, Okamoto A (2020). Microbial current production from *Streptococcus mutans* correlates with biofilm metabolic activity. Biosens. Bioelectron..

[36] Olaifa K (2021). Electroanalysis of *Candida albicans* biofilms: A suitable real-time tool for antifungal testing. Electrochim. Acta.

[37] Ajunwa OM, Odeniyi OA, Garuba EO, Marsili E, Onilude AA (2021). Influence of enhanced electrogenicity on anodic biofilm and bioelectricity production by a novel microbial consortium. Process Biochem..

[38] Doyle LE, Marsili E (2018). Weak electricigens: A new avenue for bioelectrochemical research. Bioresour. Technol..

[39] Aiyer K, Doyle LE (2022). Capturing the signal of weak electricigens: A worthy endeavour. Trends Biotechnol..

[40] Gong Z, Yu H, Zhang J, Li F, Song H (2020). Microbial electro-fermentation for synthesis of chemicals and biofuels driven by bi-directional extracellular electron transfer. Synth. Syst. Biotechnol..

[41] Kracke F, Vassilev I, Krömer JO (2015). Microbial electron transport and energy conservation—the foundation for optimizing bioelectrochemical systems. Front. Microbiol..

[42] Azeredo J (2017). Critical review on biofilm methods. Crit. Rev. Microbiol..

[43] Abás E, Aguirre-Ramírez D, Laguna M, Grasa L (2021). Selective anticancer and antimicrobial metallodrugs based on gold(Iii) dithiocarbamate complexes. Biomedicines.

