# Supplementary material for: Assessment of physiological and electrochemical effects of a repurposed zinc dithiocarbamate complex on Acinetobacter baumannii biofilms
Source: Sci Rep. 2022 Jul 9;12:11701. doi: 10.1038/s41598-022-16047-z (PMC9271062; doi:10.1038/s41598-022-16047-z)
Supplement: Supplementary file 1 — Supplementary Information. [file 41598_2022_16047_MOESM1_ESM.docx]

**Supplementary information**

**Assessment of physiological and electrochemical effects of a repurposed Zinc dithiocarbamate complex on *Acinetobacter baumannii* biofilms**

*Qing Yang^a^, Kayode Olaifa^a^, Fartisincha P. Andrew^b^, Peter A. Ajibade^c^, Obinna M. Ajunwa^a,d^, Enrico Marsili^a^*

^a^Biofilm Laboratory, Department of Chemical and Materials Engineering, School of Engineering and Digital Sciences, Nazarbayev University, 53 Kabanbay Batyr Avenue, Nur-Sultan 01000, Kazakhstan

^b^Department of Science Laboratory Technology, Modibbo Adama University, Yola, Nigeria

^c^School of Chemistry and Physics, University of KwaZulu-Natal, Scottsville, Pietermaritzburg, South Africa

^d^Department of Microbiology, Modibbo Adama University, Yola, Nigeria

Table S1: Values of zones of inhibition of *A. baumannii* by different concentrations of DTCs and DTC-Zn complexes (dashes imply no zones of inhibition) on agar diffusion assay

| Test compound | Concentrations / zones of inhibition (mm) | | | |
| --- | --- | --- | --- | --- |
|  | 5mM | 10mM | 20mM | 40mM |
| L1 | - | - | - | - |
| ZnL1 | 11.2 ±2.54 | 15 ±2.54 | 17.05 ± 4.17 | 24 ± 29 |
| L2 | - | 9.65 ± 0.35 | 10.5 ± 2.12 | 13 ± 2.83 |
| ZnL2 | 9.4 ± 0.57 | 11.5 ± 3.54 | 14.5 ± 3.54 | 15 ± 4.24 |
| L3 | - | - | - | - |
| ZnL3 | - | - | - | - |
| L4 | - | - | - | - |
| ZnL4 | - | - | - | - |

Gentamicin (10 mM) = 20.1 ± 4.38 mm

**Table S2 (A).** One-way ANOVA followed by Tukey’s test on effect of different concentrations of tested drugs leading to intracellular Protein leakage

*Overall ANOVA*

|  | DF | Sum of Squares | Mean Square | F Value | Prob>F |
| --- | --- | --- | --- | --- | --- |
| Model | 5 | 0.02717 | 0.00543 | 48.40544 | 1.53148E-7 |
| Error | 12 | 0.00135 | 1.12278E-4 |  |  |
| Total | 17 | 0.02852 |  |  |  |

*Null Hypothesis: The means of all levels are equal.*

*Alternative Hypothesis: The means of one or more levels are different.*

*At the 0.05 level, the population means are significantly different.*

*Tukey’s post hoc test for comparison of means*

|  | MeanDiff | SEM | q Value | Prob | Alpha | Sig | LCL | UCL |
| --- | --- | --- | --- | --- | --- | --- | --- | --- |
| ZnL1 12.5µM *vs.* Control | 0.02367 | 0.00865 | 3.86857 | 0.13831 | 0.05 | 0 | -0.00539 | 0.05273 |
| ZnL1 25µM *vs.* Control | 0.034 | 0.00865 | 5.55767 | 0.01909 | 0.05 | 1 | 0.00494 | 0.06306 |
| ZnL1 25µM *vs.* ZnL1 12.5µM | 0.01033 | 0.00865 | 1.6891 | 0.83145 | 0.05 | 0 | -0.01873 | 0.03939 |
| ZnL1 50µM *vs.* Control | 0.05833 | 0.00865 | 9.53522 | 2.31613E-4 | 0.05 | 1 | 0.02927 | 0.08739 |
| ZnL1 50µM *vs.* ZnL1 12.5 | 0.03467 | 0.00865 | 5.66664 | 0.01676 | 0.05 | 1 | 0.00561 | 0.06373 |
| ZnL1 50µM *vs.* ZnL1 25µM | 0.02433 | 0.00865 | 3.97755 | 0.12246 | 0.05 | 0 | -0.00473 | 0.05339 |
| ZnL1 100µM *vs.* Control | 0.12333 | 0.00865 | 20.16017 | 1.00409E-7 | 0.05 | 1 | 0.09427 | 0.15239 |
| ZnL1 100µM *vs.* ZnL1 12.5µM | 0.09967 | 0.00865 | 16.2916 | 8.25508E-7 | 0.05 | 1 | 0.07061 | 0.12873 |
| ZnL1 100 *vs.* ZnL1 25 | 0.08933 | 0.00865 | 14.6025 | 2.91801E-6 | 0.05 | 1 | 0.06027 | 0.11839 |
| ZnL1 100 µM *vs.* ZnL1 50 µM | 0.065 | 0.00865 | 10.62495 | 8.10187E-5 | 0.05 | 1 | 0.03594 | 0.09406 |
| Gen 5 µM *vs.* Control | 0.033 | 0.00865 | 5.39421 | 0.0232 | 0.05 | 1 | 0.00394 | 0.06206 |
| Gen 5 µM *vs.* ZnL1 12.5 µM | 0.00933 | 0.00865 | 1.52563 | 0.8807 | 0.05 | 0 | -0.01973 | 0.03839 |
| Gen 5 µM *vs.* ZnL1 25 µM | -1E-3 | 0.00865 | 0.16346 | 1 | 0.05 | 0 | -0.03006 | 0.02806 |
| Gen 5 µM *vs.* ZnL1 50 µM | -0.02533 | 0.00865 | 4.14101 | 0.10175 | 0.05 | 0 | -0.05439 | 0.00373 |
| Gen 5 µM *vs.* ZnL1 100 µM | -0.09033 | 0.00865 | 14.76596 | 2.57839E-6 | 0.05 | 1 | -0.11939 | -0.06127 |

* Sig equals 1 indicates that the difference of the means is significant at the 0.05 level.

Sig equals 0 indicates that the difference of the means is not significant at the 0.05 level.

**Table S2 (B).** One-way ANOVA followed by Tukey’s test on effect of different concentrations of tested drugs leading to DNA leakage

*Overall ANOVA*

|  | DF | Sum of Squares | Mean Square | F Value | Prob>F |
| --- | --- | --- | --- | --- | --- |
| Model | 5 | 2521.30558 | 504.26112 | 90.05565 | 4.33746E-9 |
| Error | 12 | 67.19327 | 5.59944 |  |  |
| Total | 17 | 2588.49885 |  |  |  |

*Null Hypothesis: The means of all levels are equal.*

*Alternative Hypothesis: The means of one or more levels are different.*

*At the 0.05 level, the population means are significantly different.*

*Tukey’s post hoc test for comparison of means*

|  | MeanDiff | SEM | q Value | Prob | Alpha | Sig | LCL | UCL |
| --- | --- | --- | --- | --- | --- | --- | --- | --- |
| ZnL1 12.5µM *vs.* Control | 8.99333 | 1.93209 | 6.58278 | 0.00568 | 0.05 | 1 | 2.50361 | 15.48306 |
| ZnL1 25µM *vs.* Control | 23.14 | 1.93209 | 16.93759 | 5.05764E-7 | 0.05 | 1 | 16.65027 | 29.62973 |
| ZnL1 25µM *vs.* ZnL1 12.5µM | 14.14667 | 1.93209 | 10.35482 | 1.04454E-4 | 0.05 | 1 | 7.65694 | 20.63639 |
| ZnL1 50µM *vs.* Control | 30.95333 | 1.93209 | 22.65666 | 9.96348E-7 | 0.05 | 1 | 24.46361 | 37.44306 |
| ZnL1 50µM *vs.* ZnL1 12.5 | 21.96 | 1.93209 | 16.07388 | 9.71205E-7 | 0.05 | 1 | 15.47027 | 28.44973 |
| ZnL1 50µM *vs.* ZnL1 25µM | 7.81333 | 1.93209 | 5.71906 | 0.01574 | 0.05 | 1 | 1.32361 | 14.30306 |
| ZnL1 100µM *vs.* Control | 32.34667 | 1.93209 | 23.67652 | 2.3826E-7 | 0.05 | 1 | 25.85694 | 38.83639 |
| ZnL1 100µM *vs.* ZnL1 12.5µM | 23.35333 | 1.93209 | 17.09375 | 4.19133E-7 | 0.05 | 1 | 16.86361 | 29.84306 |
| ZnL1 100 *vs.* ZnL1 25 | 9.20667 | 1.93209 | 6.73893 | 0.00474 | 0.05 | 1 | 2.71694 | 15.69639 |
| ZnL1 100 µM *vs.* ZnL1 50 µM | 1.39333 | 1.93209 | 1.01987 | 0.97545 | 0.05 | 0 | -5.09639 | 7.88306 |
| Gen 5 µM *vs.* Control | 12.05667 | 1.93209 | 8.82502 | 4.77163E-4 | 0.05 | 1 | 5.56694 | 18.54639 |
| Gen 5 µM *vs.* ZnL1 12.5 µM | 3.06333 | 1.93209 | 2.24224 | 0.62178 | 0.05 | 0 | -3.42639 | 9.55306 |
| Gen 5 µM *vs.* ZnL1 25 µM | -11.08333 | 1.93209 | 8.11258 | 0.00102 | 0.05 | 1 | -17.57306 | -4.59361 |
| Gen 5 µM *vs.* ZnL1 50 µM | -18.89667 | 1.93209 | 13.83164 | 5.28216E-6 | 0.05 | 1 | -25.38639 | -12.40694 |
| Gen 5 µM *vs.* ZnL1 100 µM | -20.29 | 1.93209 | 14.8515 | 2.4173E-6 | 0.05 | 1 | -26.77973 | -13.80027 |

* Sig equals 1 indicates that the difference of the means is significant at the 0.05 level.

Sig equals 0 indicates that the difference of the means is not significant at the 0.05 level.

**Table S2 (C).** One-way ANOVA followed by Tukey’s test on effect of different concentrations of tested drugs leading to ATP synthase loss.

*Overall ANOVA*

| DF | Sum of Squares | Mean Square | F Value | Prob>F |  |
| --- | --- | --- | --- | --- | --- |
| Model | 5 | 3.90944E-4 | 7.81889E-5 | 41.39412 | 3.69542E-7 |
| Error | 12 | 2.26667E-5 | 1.88889E-6 |  |  |
| Total | 17 | 4.13611E-4 |  |  |  |

*Null Hypothesis: The means of all levels are equal.*

*Alternative Hypothesis: The means of one or more levels are different.*

*At the 0.05 level, the population means are significantly different.*

*Tukey’s post hoc test for comparison of means*

|  | MeanDiff | SEM | q Value | Prob | Alpha | Sig | LCL | UCL |
| --- | --- | --- | --- | --- | --- | --- | --- | --- |
| ZnL1 12.5µM *vs.* Control | -0.00567 | 0.00112 | 7.14143 | 0.00299 | 0.05 | 1 | -0.00944 | -0.0019 |
| ZnL1 25µM *vs.* Control | -0.00967 | 0.00112 | 12.18244 | 2.02548E-5 | 0.05 | 1 | -0.01344 | -0.0059 |
| ZnL1 25µM *vs.* ZnL1 12.5µM | -0.004 | 0.00112 | 5.04101 | 0.03539 | 0.05 | 1 | -0.00777 | -2.3073E-4 |
| ZnL1 50µM *vs.* Control | -0.011 | 0.00112 | 13.86277 | 5.15519E-6 | 0.05 | 1 | -0.01477 | -0.00723 |
| ZnL1 50µM *vs.* ZnL1 12.5 | -0.00533 | 0.00112 | 6.72134 | 0.00484 | 0.05 | 1 | -0.0091 | -0.00156 |
| ZnL1 50µM *vs.* ZnL1 25µM | -0.00133 | 0.00112 | 1.68034 | 0.83429 | 0.05 | 0 | -0.0051 | 0.00244 |
| ZnL1 100µM *vs.* Control | -0.015 | 0.00112 | 18.90378 | 1.81965E-7 | 0.05 | 1 | -0.01877 | -0.01123 |
| ZnL1 100µM *vs.* ZnL1 12.5µM | -0.00933 | 0.00112 | 11.76235 | 2.90796E-5 | 0.05 | 1 | -0.0131 | -0.00556 |
| ZnL1 100 *vs.* ZnL1 25 | -0.00533 | 0.00112 | 6.72134 | 0.00484 | 0.05 | 1 | -0.0091 | -0.00156 |
| ZnL1 100 µM *vs.* ZnL1 50 µM | -0.004 | 0.00112 | 5.04101 | 0.03539 | 0.05 | 1 | -0.00777 | -2.3073E-4 |
| Gen 5 µM *vs.* Control | -0.009 | 0.00112 | 11.34227 | 4.21157E-5 | 0.05 | 1 | -0.01277 | -0.00523 |
| Gen 5 µM *vs.* ZnL1 12.5 µM | -0.00333 | 0.00112 | 4.20084 | 0.09501 | 0.05 | 0 | -0.0071 | 4.35937E-4 |
| Gen 5 µM *vs.* ZnL1 25 µM | 6.66667E-4 | 0.00112 | 0.84017 | 0.98949 | 0.05 | 0 | -0.0031 | 0.00444 |
| Gen 5 µM *vs.* ZnL1 50 µM | 0.002 | 0.00112 | 2.5205 | 0.50991 | 0.05 | 0 | -0.00177 | 0.00577 |
| Gen 5 µM *vs.* ZnL1 100 µM | 0.006 | 0.00112 | 7.56151 | 0.00186 | 0.05 | 1 | 0.00223 | 0.00977 |

* Sig equals 1 indicates that the difference of the means is significant at the 0.05 level.

Sig equals 0 indicates that the difference of the means is not significant at the 0.05 level.

**Table S2 (D).** One-way ANOVA followed by Tukey’s test on effect of different concentrations of tested drugs leading to potassium ion leakage.

*Overall ANOVA*

|  | DF | Sum of Squares | Mean Square | F Value | Prob>F |
| --- | --- | --- | --- | --- | --- |
| Model | 5 | 0.00316 | 6.32722E-4 | 116.21429 | 9.8011E-10 |
| Error | 12 | 6.53333E-5 | 5.44444E-6 |  |  |
| Total | 17 | 0.00323 |  |  |  |

*Tukey’s post hoc test for comparison of means*

|  | MeanDiff | SEM | q Value | Prob | Alpha | Sig | LCL | UCL |
| --- | --- | --- | --- | --- | --- | --- | --- | --- |
| ZnL1 12.5µM *vs.* Control | 0.02867 | 0.00191 | 21.27948 | 2.55743E-7 | 0.05 | 1 | 0.02227 | 0.03507 |
| ZnL1 25µM *vs.* Control | 0.03533 | 0.00191 | 26.2282 | 0 | 0.05 | 1 | 0.02893 | 0.04173 |
| ZnL1 25µM *vs.* ZnL1 12.5µM | 0.00667 | 0.00191 | 4.94872 | 0.0395 | 0.05 | 1 | 2.67391E-4 | 0.01307 |
| ZnL1 50µM *vs.* Control | 0.03567 | 0.00191 | 26.47563 | 0 | 0.05 | 1 | 0.02927 | 0.04207 |
| ZnL1 50µM *vs.* ZnL1 12.5 | 0.007 | 0.00191 | 5.19615 | 0.0294 | 0.05 | 1 | 6.00724E-4 | 0.0134 |
| ZnL1 50µM *vs.* ZnL1 25µM | 3.33333E-4 | 0.00191 | 0.24744 | 0.99997 | 0.05 | 0 | -0.00607 | 0.00673 |
| ZnL1 100µM *vs.* Control | 0.03633 | 0.00191 | 26.97051 | 0 | 0.05 | 1 | 0.02993 | 0.04273 |
| ZnL1 100µM *vs.* ZnL1 12.5µM | 0.00767 | 0.00191 | 5.69102 | 0.01628 | 0.05 | 1 | 0.00127 | 0.01407 |
| ZnL1 100 *vs.* ZnL1 25 | 1E-3 | 0.00191 | 0.74231 | 0.99401 | 0.05 | 0 | -0.0054 | 0.0074 |
| ZnL1 100 µM *vs.* ZnL1 50 µM | 6.66667E-4 | 0.00191 | 0.49487 | 0.99911 | 0.05 | 0 | -0.00573 | 0.00707 |
| Gen 5 µM *vs.* Control | 0.03767 | 0.00191 | 27.96025 | 0 | 0.05 | 1 | 0.03127 | 0.04407 |
| Gen 5 µM *vs.* ZnL1 12.5 µM | 0.009 | 0.00191 | 6.68077 | 0.00507 | 0.05 | 1 | 0.0026 | 0.0154 |
| Gen 5 µM *vs.* ZnL1 25 µM | 0.00233 | 0.00191 | 1.73205 | 0.8172 | 0.05 | 0 | -0.00407 | 0.00873 |
| Gen 5 µM *vs.* ZnL1 50 µM | 0.002 | 0.00191 | 1.48461 | 0.89172 | 0.05 | 0 | -0.0044 | 0.0084 |
| Gen 5 µM *vs.* ZnL1 100 µM | 0.00133 | 0.00191 | 0.98974 | 0.97841 | 0.05 | 0 | -0.00507 | 0.00773 |

* Sig equals 1 indicates that the difference of the means is significant at the 0.05 level.

Sig equals 0 indicates that the difference of the means is not significant at the 0.05 level.

**Table S3 (A).** One-way ANOVA followed by Tukey’s test on effects of ZnL1 in comparison with Gen on forming biofilm – biofilm inhibition.

*Overall ANOVA*

|  | DF | Sum of Squares | Mean Square | F Value | Prob>F |
| --- | --- | --- | --- | --- | --- |
| Model | 6 | 58886.84228 | 9814.47371 | 126.11337 | 0 |
| Error | 28 | 2179.03361 | 77.82263 |  |  |
| Total | 34 | 61065.87589 |  |  |  |

*Null Hypothesis: The means of all levels are equal.*

*Alternative Hypothesis: The means of one or more levels are different.*

*At the 0.05 level, the population means are significantly different.*

*Tukey’s post hoc test for comparison of means*

|  | MeanDiff | SEM | q Value | Prob | Alpha | Sig | LCL | UCL |
| --- | --- | --- | --- | --- | --- | --- | --- | --- |
| ZnL1 50µM *vs.* ZnL1 12.5 µM | 103.2088 | 5.57934 | 26.16067 | 1.64461E-7 | 0.05 | 1 | 85.51038 | 120.90722 |
| ZnL1 100 µM *vs.* ZnL1 12.5 µM | 103.77655 | 5.57934 | 26.30457 | 1.65845E-7 | 0.05 | 1 | 86.07813 | 121.47497 |
| ZnL1 100 µM *vs.* ZnL1 50 µM | 0.56775 | 5.57934 | 0.14391 | 1 | 0.05 | 0 | -17.13067 | 18.26617 |
| ZnL1 200 µM *vs.* ZnL1 12.5 µM | 105.03371 | 5.57934 | 26.62323 | 4.94028E-7 | 0.05 | 1 | 87.33529 | 122.73213 |
| ZnL1 200 µM *vs.* ZnL1 50 µM | 1.82491 | 5.57934 | 0.46257 | 0.99988 | 0.05 | 0 | -15.87351 | 19.52333 |
| ZnL1 200 µM *vs.* ZnL1 100 µM | 1.25716 | 5.57934 | 0.31866 | 0.99999 | 0.05 | 0 | -16.44126 | 18.95558 |
| ZnL1 400 µM *vs.* ZnL1 12.5 µM | 93.75982 | 5.57934 | 23.7656 | 2.68557E-8 | 0.05 | 1 | 76.0614 | 111.45824 |
| ZnL1 400 µM *vs.* ZnL1 50 µM | -9.44898 | 5.57934 | 2.39506 | 0.62563 | 0.05 | 0 | -27.1474 | 8.24944 |
| ZnL1 400 µM *vs.* ZnL1 100 µM | -10.01673 | 5.57934 | 2.53897 | 0.56181 | 0.05 | 0 | -27.71515 | 7.68169 |
| ZnL1 400 µM *vs.* ZnL1 200 µM | -11.27389 | 5.57934 | 2.85763 | 0.42473 | 0.05 | 0 | -28.97231 | 6.42453 |
| Gen 5 µM *vs.* ZnL1 12.5 µM | 38.48532 | 5.57934 | 9.755 | 3.29196E-6 | 0.05 | 1 | 20.7869 | 56.18375 |
| Gen 5 µM *vs.* ZnL1 50 µM | -64.72348 | 5.57934 | 16.40567 | 2.99508E-8 | 0.05 | 1 | -82.4219 | -47.02505 |
| Gen 5 µM *vs.* ZnL1 100 µM | -65.29123 | 5.57934 | 16.54958 | 2.93898E-8 | 0.05 | 1 | -82.98965 | -47.5928 |
| Gen 5 µM *vs.* ZnL1 200 µM | -66.54839 | 5.57934 | 16.86823 | 2.8164E-8 | 0.05 | 1 | -84.24681 | -48.84996 |
| Gen 5 µM µM *vs.* ZnL1 400 µM | -55.2745 | 5.57934 | 14.0106 | 4.17912E-8 | 0.05 | 1 | -72.97292 | -37.57607 |
| Gen 50 µM *vs.* ZnL1 12.5 µM | 121.70122 | 5.57934 | 30.848 | 0 | 0.05 | 1 | 104.0028 | 139.39964 |
| Gen 50 µM *vs.* ZnL1 50 µM | 18.49242 | 5.57934 | 4.68733 | 0.03616 | 0.05 | 1 | 0.794 | 36.19084 |
| Gen 50 µM*vs.* ZnL1 100 µM | 17.92467 | 5.57934 | 4.54342 | 0.04563 | 0.05 | 1 | 0.22625 | 35.62309 |
| Gen 50 µM *vs.* ZnL1 200 µM | 16.66751 | 5.57934 | 4.22477 | 0.07502 | 0.05 | 0 | -1.03091 | 34.36593 |
| Gen 50 µM *vs.* ZnL1 400 µM | 27.9414 | 5.57934 | 7.0824 | 4.87135E-4 | 0.05 | 1 | 10.24298 | 45.63982 |
| Gen 50 µM *vs.* Gen 5 µM | 83.2159 | 5.57934 | 21.093 | 0 | 0.05 | 1 | 65.51748 | 100.91432 |

* Sig equals 1 indicates that the difference of the means is significant at the 0.05 level.

Sig equals 0 indicates that the difference of the means is not significant at the 0.05 level.

**Table S3 (B).** One-way ANOVA followed by Tukey’s test on effects of ZnL1 in comparison with Gen on pre-formed biofilm – biofilm removal.

*Overall ANOVA*

|  | DF | Sum of Squares | Mean Square | F Value | Prob>F |
| --- | --- | --- | --- | --- | --- |
| Model | 5 | 4564.18554 | 912.83711 | 30.45892 | 2.02056E-6 |
| Error | 12 | 359.6334 | 29.96945 |  |  |
| Total | 17 | 4923.81894 |  |  |  |

*Null Hypothesis: The means of all levels are equal.*

*Alternative Hypothesis: The means of one or more levels are different.*

*At the 0.05 level, the population means are significantly different.*

| MeanDiff | SEM | q Value | Prob | Alpha | Sig | LCL | UCL |  |
| --- | --- | --- | --- | --- | --- | --- | --- | --- |
| ZnL1 100 µM *vs.* ZnL1 50 µM | -1.76144 | 4.46986 | 0.5573 | 0.99844 | 0.05 | 0 | -16.77534 | 13.25245 |
| ZnL1 125 µM *vs.* ZnL1 50 µM | 4.33353 | 4.46986 | 1.37108 | 0.91916 | 0.05 | 0 | -10.68037 | 19.34742 |
| ZnL1 125 µM *vs.* ZnL1 100 µM | 6.09497 | 4.46986 | 1.92838 | 0.7465 | 0.05 | 0 | -8.91893 | 21.10887 |
| ZnL1 250 µM *vs.* ZnL1 50 µM | 4.25574 | 4.46986 | 1.34647 | 0.92451 | 0.05 | 0 | -10.75816 | 19.26963 |
| ZnL1 250 µM *vs.* ZnL1 100 µM | 6.01718 | 4.46986 | 1.90377 | 0.7558 | 0.05 | 0 | -8.99672 | 21.03108 |
| ZnL1 250 µM *vs.* ZnL1 125 µM | -0.07779 | 4.46986 | 0.02461 | 1 | 0.05 | 0 | -15.09169 | 14.93611 |
| Gen 25 µM *vs.* ZnL1 50 µM | 16.27838 | 4.46986 | 5.1503 | 0.03106 | 0.05 | 1 | 1.26449 | 31.29228 |
| Gen 25 µM *vs.* ZnL1 100 µM | 18.03983 | 4.46986 | 5.7076 | 0.01596 | 0.05 | 1 | 3.02593 | 33.05372 |
| Gen 25 µM *vs.* ZnL1 125 µM | 11.94486 | 4.46986 | 3.77922 | 0.15264 | 0.05 | 0 | -3.06904 | 26.95875 |
| Gen 25 µM *vs.* ZnL1 250 µM | 12.02265 | 4.46986 | 3.80383 | 0.14857 | 0.05 | 0 | -2.99125 | 27.03654 |
| DMSO *vs.* ZnL1 50 µM | -35.2264 | 4.46986 | 11.14524 | 5.02731E-5 | 0.05 | 1 | -50.2403 | -20.2125 |
| DMSO *vs.* ZnL1 100 µM | -33.46495 | 4.46986 | 10.58794 | 8.38685E-5 | 0.05 | 1 | -48.47885 | -18.45106 |
| DMSO *vs.* ZnL1 125 µM | -39.55993 | 4.46986 | 12.51632 | 1.52871E-5 | 0.05 | 1 | -54.57382 | -24.54603 |
| DMSO *vs.* ZnL1 250 µM | -39.48214 | 4.46986 | 12.49171 | 1.56048E-5 | 0.05 | 1 | -54.49603 | -24.46824 |
| DMSO *vs.* Gen 25 µM | -51.50478 | 4.46986 | 16.29554 | 8.23075E-7 | 0.05 | 1 | -66.51868 | -36.49089 |

*Tukey’s post hoc test for comparison of means*

* Sig equals 1 indicates that the difference of the means is significant at the 0.05 level.

Sig equals 0 indicates that the difference of the means is not significant at the 0.05 level.


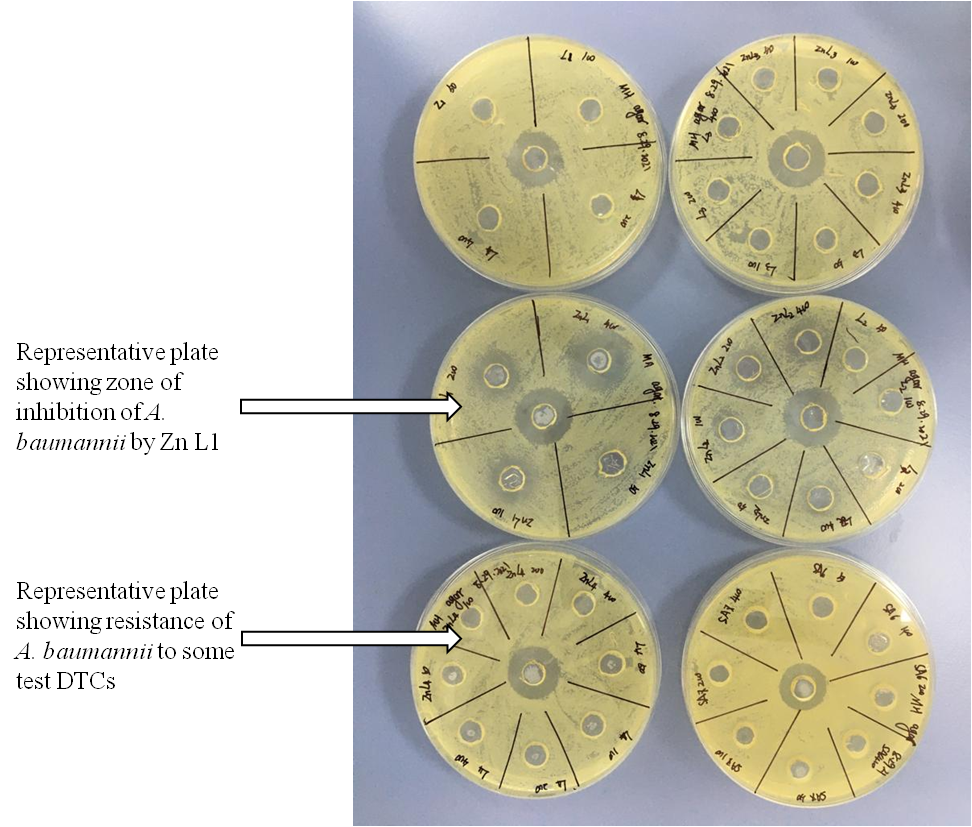


A


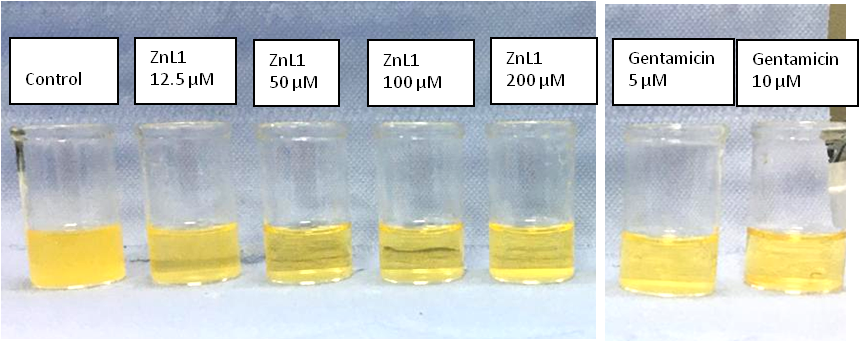


B

Figure S1: Representative solid media (A) and liquid media (B) drug susceptibility tests of ZnL1 on *A. baumannnii* in comparison with gentamicin. **Note**: Gentamicin (10 mM) controls were placed at centers of all agar plates in diffusion experiments above.


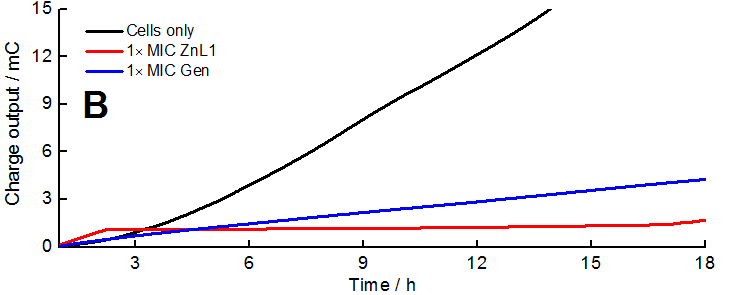


Figure S2: Charge output of *A. baumannii* planktonic cells treated with ZnL1 in comparison with cells treated with gentamicin and untreated controls at 18 h.





Figure S 3: Differential pulse voltammetry analyses of *A. baumannii* biofilm treated with ZnL1 at sub-inhibitory concentrations (0.1, 1, 5 and 10 µM) with up to 48/72 hours exposure. As concentration increased, the signature peak of *A. baumannii* biofilms at approximately 0 – 0.15V gradually disappears. Most prominent peaks were observed as the smallest concentration of ZnL1 used (0.1 µM) was applied. This proves that ZnL1 altered respiratory mechanisms of *A. baumannii* thereby inducing cell death.
